# Sequential change detection and monitoring of temporal trends in random‐effects meta‐analysis

**DOI:** 10.1002/jrsm.1222

**Published:** 2016-12-08

**Authors:** Samson Henry Dogo, Allan Clark, Elena Kulinskaya

**Affiliations:** ^1^ School of Computing SciencesUniversity of East AngliaNorwichUK

**Keywords:** sequential meta‐analysis, cumulative meta‐analysis, CUSUM, bootstrap

## Abstract

Temporal changes in magnitude of effect sizes reported in many areas of research are a threat to the credibility of the results and conclusions of meta‐analysis. Numerous sequential methods for meta‐analysis have been proposed to detect changes and monitor trends in effect sizes so that meta‐analysis can be updated when necessary and interpreted based on the time it was conducted. The difficulties of sequential meta‐analysis under the random‐effects model are caused by dependencies in increments introduced by the estimation of the heterogeneity parameter *τ* ^2^. In this paper, we propose the use of a retrospective cumulative sum (CUSUM)‐type test with bootstrap critical values. This method allows retrospective analysis of the past trajectory of cumulative effects in random‐effects meta‐analysis and its visualization on a chart similar to CUSUM chart. Simulation results show that the new method demonstrates good control of Type I error regardless of the number or size of the studies and the amount of heterogeneity. Application of the new method is illustrated on two examples of medical meta‐analyses. © 2016 The Authors. *Research Synthesis Methods* published by John Wiley & Sons Ltd.

## 1 Introduction

Meta‐analysis is a statistical technique used to combine results from related but independent studies in order to provide an estimate of the overall treatment effect. In clinical applications, it is often used to synthesize and strengthen evidence about treatment efficacy or harm and to provide evidence for decision making. It has become increasingly important with the increasing number of clinical studies providing sometimes inconclusive and inconsistent results. By combining information from different studies, meta‐analysis increases the overall sample size and achieves a higher statistical power for the outcome of interest compared with individual studies.

However, recent findings in many areas of research have shown that effect size estimates used in meta‐analyses may change significantly over time. For example, Hodgson *et al.* (1989) found a decline of about 1.4% per annum in the sensitivity of chest X‐rays in detecting hypersensitivity pneumonitis, which they attributed to secular trends in knowledge and earlier diagnosis or changes in the disease itself. Nieuwkamp *et al.* (2009) found a decrease in case fatality of aneurysmal sub‐arachnoid haemorrhage during the period 1960–1995, which they attributed to improvement in early diagnostic and treatment strategies. Similar temporal changes have been reported in education (Hyde *et al.,* 1990), medicine (Gehr *et al.,* 2006), psychology (Brugger *et al.,* 2011; Twenge *et al.,* 2008; Grabe *et al.,* 2008), to mention but a few. These changes in effect sizes can be dramatic and often lead to the loss or gain of statistical significance (Kulinskaya and Koricheva, 2010). Therefore, if meta‐analysis is conducted ignoring temporal changes, when such changes are actually present, its results and conclusions are likely to be misleading. In case of a monotonic temporal trend, meta‐regression with time as a covariate can be used to evaluate such a trend and to adjust for it, see Shi and Copas (2004); Baker and Jackson (2010).

We consider a different although related problem of sequential monitoring of changes in effect size estimates in meta‐analysis (Lau *et al.,* 1992; Leimu and Koricheva, 2004; Pogue and Yusuf, 1997; Wetterslev *et al.,* 2008; Higgins *et al.,* 2011; Whitehead, 1997b; Bollen *et al.,* 2006; Kulinskaya and Koricheva, 2010; Lan *et al.,* 2003). These methods are aimed at gauging sufficiency of evidence (Lau *et al.,* 1992; Pogue and Yusuf, 1997; Wetterslev *et al.,* 2008) or at monitoring effect size estimates (Leimu and Koricheva, 2004; Kulinskaya and Koricheva, 2010; Ioannidis and Trikalinos, 2005). However, these methods were largely derived for fixed effect model (FEM), with or without further empirical corrections for random‐effects model (REM). In REM, the analysis incorporates the heterogeneity variance, *τ* ^2^_,_ and its estimation creates dependency in consecutively estimated cumulative effects which violates the assumed independence of increments in sequential methods.

In Dogo *et al.* (2015), we introduced the use of a (Gombay and Serban, 2005) truncated CUSUM‐type test (Gombay method) for sequential random‐effects meta‐analysis. For large within‐study sample sizes, the Gombay method is valid under the random‐effects model of meta‐analysis. However, the critical values for the Gombay test are derived from asymptotic theory, and our simulations (Dogo *et al.,* 2015) demonstrated that the test does not control the Type I error satisfactorily.

In the current paper, we review the existing sequential methods for meta‐analysis and propose the use of bootstrap‐based critical values for use with the Gombay method. This results in a method allowing retrospective analysis of the past trajectory of cumulative effects in random‐effects meta‐analysis and its visualization on a chart similar to a CUSUM chart. The proposed method constitutes a useful tool for retrospective monitoring of effect size estimates. The rest of the paper is organized as follows. In Section 2, we review the existing sequential methods for meta‐analysis. In Section 3, we formulate the Gombay test statistic for random effects model of meta‐analysis. In Section 4, we provide the algorithm to obtain the bootstrap critical values for the random‐effects model. In Section 5, we report on a simulation study to evaluate the performance of the new method. In Section 6, we demonstrate the application of the new method to two examples of medical meta‐analyses. Section 7 is the summary and conclusions.

## 2 Existing sequential methods for monitoring temporal changes in effect sizes in meta‐analysis

In this Section, we recap the fixed effect and the random effects models of meta‐analysis, summarize four existing methods of estimation of the between‐studies variance component *τ* ^2^ before reviewing the existing sequential methods for monitoring effect sizes.

### 2.1 Fixed effect and random effects models

To combine the results from K studies, the two main models are the FEM and the REM. The fixed effect model assumes that all the included studies investigate the same population and therefore share a common location parameter. Denote by *y*_1_, *y*_2_, …, *y*_*K*_ the estimates of treatment effects derived from K studies. When *y*_*i*_'s are sample means or mean differences, the fixed effect model is given by

(1) $$y_{i}=\theta +e_{i}\text{,}$$

where *θ* is the common location parameter, $e_{i}∼N\left(0,\sigma_{i}^{2}\right)$ are the sampling errors, $\sigma_{i}^{2}$ are the within‐study variances, for *i* = 1, ⋯, *K*. For other effect measures, approximate normality of *y*_*i*_'s holds when the sample sizes *n*_*i*_ of the studies are reasonably large. Appropriate estimates of the variances $\sigma_{i}^{2}$ are easily calculated for all effect measures used in meta‐analysis and, for large within study sample sizes, can be treated as known constants (Viechtbauer, 2007). In FEM, each study is assigned a weight proportional to the inverse of the within‐study variance, which is denoted by $w_{i}=1/\sigma_{i}^{2}$. The combined effect is estimated as a weighted mean of the individual effect estimates given by

(2) $${\hat{\theta }}_{FEM}=\sum_{i=1}^{K}\;w_{i}y_{i}/W_{K}\text{,}$$

where $W_{K}=\sum_{i=1}^{K}\;w_{i}$. The variance of the combined effect is given by the inverse of the sum of weights, $W_{K}^{-1}$. Standard inference in FEM is based on approximate normality of the distribution of the combined effect, ${\hat{\theta }}_{FEM}∼N\left(\theta ,W_{K}^{-1}\right)$.

Cochran's Q statistic

(3) $$Q=\sum_{i=1}^{K}\;w_{i}{\left(y_{i}-{\hat{\theta }}_{FEM}\right)}^{2}$$

plays an important role in meta‐analysis. It is widely used in inference on heterogeneity of treatment effects. The Q statistic is routinely assumed to follow the chi‐square distribution with K‐1 degrees of freedom, $\chi_{K-1}^{2}$, although this is true only for very large sample sizes, see Hoaglin (2016) for details.

Random effects model is generally preferred to the fixed effect model (Hunter and Schmidt, 2000) because of its ability to account for variation in effects across the studies. Random effects model allows different mean effects *θ*_*i*_ across the studies and it assumes that they are sampled from a population of parameters with mean *θ*. REM is a two‐level model given by

(4) yi=θi+ei;ei∼N0σi2;θi=θ+εi;εi∼N0τ2,

where $\sigma_{i}^{2}$ and *τ* ^2^ are the within‐study and between‐study variances, respectively.

Marginally, the random effects model is defined by

(5) $$y_{i}=\theta +\xi_{i};\;\xi_{i}∼N\left(0,\tau^{2}+\sigma_{i}^{2}\right)\text{.}$$

The between‐study variance, *τ* ^2^, describes the degree of heterogeneity among the effect estimates. The special case where *τ* ^2^ = 0 implies that the effect sizes, *θ*_1_ = *θ*_2_ = … = *θ*_*K*_, are homogeneous, and the resulting model reduces to FEM in Equation (1). The weights assigned to studies in REM are inverse variance weights defined by $w_{i}^{*}=w_{i}\left(\tau^{2}\right)={\left(\tau^{2}+\sigma_{i}^{2}\right)}^{-1}$. Estimated values of *τ* ^2^ and $\sigma_{i}^{2}$ are substituted in practice. Similar to FEM, the combined effect in REM is estimated as a weighted mean of the individual effect estimates, ${\hat{\theta }}_{REM}=\sum_{i}\;w_{i}^{*}y_{i}/W_{K}^{*}$, where $W_{K}^{*}=\sum_{i=1}^{K}\;w_{i}^{*}$. Once more, typically the inference is based on the approximate normality of the combined effect.

Estimation of the between‐study variance, *τ* ^2^ plays a crucial role in REM. There exist a number of methods for estimating *τ* ^2^, (Veroniki *et al.,* 2016), but we describe here only the most commonly used methods by DerSimonian and Laird (1986), Mandel and Paule (1970), and the restricted maximum likelihood (REML) method along with the method by Higgins *et al.* (2011) proposed specifically for sequential testing in meta‐analysis. Each of these methods differs in terms of precision and bias in estimating *τ* ^2^, and in Section 4, we examine by simulation how this affects the sequential testing.

**DerSimonian and Laird (** **1986** **) method**

The DerSimonian and Laird (1986) estimator is given by

(6) $${\hat{\tau }}_{DL}^{2}=\text{max}\left(\frac{Q-\left(K-1\right)}{W_{K}-\sum_{i=1}^{K}\;w_{i}^{2}/W_{K}},\;0\right)\text{.}$$

**Higgins *et al.* (** **2011** **) method**

The Higgins *et al.* (2011) estimator is a modification of the DerSimonian and Laird (1986) method using semi‐Bayes approach. It is defined by

(7) $${\hat{\tau }}_{H}^{2}=\frac{2\lambda +K{\hat{\tau }}_{DL}^{2}}{2\eta +K-2}\text{,}$$

where *λ* and *η* are parameters of an inverse gamma prior distribution for *τ* ^2^.

**Mandel and Paule (** **1970** **) method**

The Mandel and Paule (1970) estimator denoted by ${\hat{\tau }}_{MP}^{2}$ (see also Paule and Mandel (1982)) is calculated from the solution of the estimating equation for the expected value of the *Q*(*τ* ^2^) statistic given by

(8) $$Q\left(\tau^{2}\right)=\sum_{i=1}^{K}\;w_{i}\left(\tau^{2}\right){\left(y_{i}-\hat{\theta }\left(\tau^{2}\right)\right)}^{2}\text{,}$$

where $\hat{\theta }\left(\tau^{2}\right)$ and *w*_*i*_(*τ* ^2^) are functions of *τ* ^2^. For known variances $\sigma_{i}^{2}$ and *τ* ^2^, the *Q*(*τ* ^2^) statistic has the chi‐square distribition with K‐1 degrees of freedom, and the Mandel–Paule estimator ${\hat{\tau }}_{MP}^{2}$ is found from the estimating equation $Q\left({\hat{\tau }}_{MP}^{2}\right)=K-1$, if the solution exists. If *Q*(0) < *K* − 1, we set ${\hat{\tau }}_{MP}^{2}=0$.

**Restricted maximum likelihood method**

The restricted maximum likelihood estimator of *τ* ^2^ is given by an iterative solution of the equation

(9) $${\hat{\tau }}_{\text{REML}}^{2}=\text{max}\left(\frac{\sum_{i=1}^{K}\;w_{i}^{*2}\left[{\left(y_{i}-\hat{\theta }\right)}^{2}-\sigma_{i}^{2}\right]}{\sum_{i=1}^{K}\;w_{i}^{*2}}+\frac{1}{\sum_{i=1}^{K}\;w_{i}^{*}},\;0\right)\text{.}$$

### 2.2 Sequential methods in meta‐analysis

Several methods have been proposed for sequential monitoring of temporal trends in meta‐analysis. Historically, the first method proposed by Lau *et al.* (1992) was cumulative meta‐analysis (CMA), which can be described as an open sequential test. The method involves pooling effect size estimates in a cumulative manner as new trial results are published. Lau *et al.* (1992) had proposed the use of the method for monitoring interventions across several randomized controlled trials, with the goal to understanding when evidence becomes definitive. CMA is routinely used for monitoring temporal changes in effect sizes, see Lau *et al.* (1992); Ioannidis and Trikalinos (2005); Leimu and Koricheva (2004). However, CMA involves repeated analysis of the accumulating evidence, and thus, even if there is no treatment effect, the multiple testing involved leads to the inflation of Type I error.

A second group of methods is the sequential meta‐analysis (SMA). These methods involve the use of formal group sequential boundaries to monitor CMA and were proposed by Pogue and Yusuf (1997) to address the issue of inflated Type I error in CMA. SMA involves calculation of an optimum information size (OIS) and then determines the monitoring boundaries using an alpha spending function (Lan and DeMets, 1983) and stochastic curtailment. However, the calculation of the OIS is based on a fixed effect model, and hence, the method is only appropriate for FEM. A number of methods were developed to correct for this. Wetterslev *et al.* (2008, 2009) used a heterogeneity inflated OIS, but this method is problematic (Kulinskaya and Wood, 2013). Whitehead (1997a) describes the use of the standard stopping boundaries for random‐effects meta‐analysis. Bollen *et al.* (2006) used the double triangular test in a retrospective meta‐analysis. Higgins *et al.* (2011) proposed a sequential method for random‐effects meta‐analysis that uses a semi‐Bayes procedure to update evidence on the between‐study variance, starting with an informative prior distribution that may be based on findings from a previous meta‐analysis. A common issue for these methods is that the monitoring boundaries are generally defined based on FEM and do not incorporate the presence of heterogeneity in treatment effects. As a result, as revealed by simulations, these methods lead to a considerable inflation of the Type I error when the values of *τ* ^2^ are large, Higgins *et al.* (2011); Wetterslev *et al.* (2008).

A third group of methods involves the ‘penalized *Z*‐test’ introduced by Lan *et al.* (2003). This is an alternative approach to address the issue of inflated Type I error in CMA. The method is based on the law of iterated logarithm to ‘penalize’ for the multiple testing in CMA. The usual Wald‐test statistic for significance of the combined effect at the k‐th interim analysis is adjusted by a constant factor and is defined by

(10) $$Z^{*}\left(k\right)=\frac{\sum_{1}^{k}\;w_{i}^{*}y_{i}}{\sqrt{\lambda W_{k}^{*}\text{ln ln}\left(W_{k}\right)}}\text{,}$$

where *λ* is the adjustment factor determined using simulation. Lan *et al.* (2003) claim that the penalized *Z*‐test exhibits a good control of the Type I error in CMA both in FEM and REM when a reasonable value of *λ* is used. For example, the value of *λ* = 1.5 was found to control the Type I error in FEM, while the value of *λ* = 2 was found to control the Type I error in REM when relative risks, odds ratios or risks differences were used as effect measures and meta‐analyses included up to 25 studies (Hu *et al.,* 2007). The choice of *λ* is important in controlling the Type I error; however, its value varies according to the type of effect measure, number of studies, average study size and the amount of heterogeneity in the treatment effects. Therefore, the determination of the ‘reasonable value of *λ*’ can be difficult in practice.

Recently, Kulinskaya and Koricheva (2010) proposed the use of quality control charts for detection of outliers and temporal trends in meta‐analysis. The use of QC charts in meta‐analysis is straightforward if the sequential effect estimates are independent and their distribution can be approximated by the normal distribution. This is true in FEM, but in random effects model, the estimation of *τ* ^2^ introduces dependency between the sequential effect estimates, and hence, their distribution is not consistent with the standard assumptions of the QC charts.

In this paper, we propose the use of Gombay (2003) truncated CUSUM‐type test statistic with critical values estimated by the bootstrap. The between‐study variance component *τ* ^2^ is treated as a nuisance parameter, and it is included in the determination of the bootstrap critical values.

## 3 Formulation of Gombay test statistic for random effects model

In this Section, we describe the Gombay test (Gombay, 2003) in its generality before formulating the Gombay test statistic for sequential random‐effects meta‐analysis.

### 3.1 Gombay test

The Gombay test described below was introduced as test II in Gombay (2003). It is a sequential change detection test for parametric models in the presence of a vector nuisance parameter. Consider a sequence of independent random variables (r.v.) *X*_1_, *X*_2_, …, $∼f_{\theta_{i},\eta_{i}}$, where f is a probability density function, *θ* is a (vector) parameter of interest and *η* is a nuisance parameter. Consider a test for the composite hypothesis $H_{0}:\theta_{i}=\theta_{0},\;\eta_{i}=\eta ;\;i=1,\;2,\;\ldots \text{against alternatives}\;H_{1r}:\left\{\begin{array}{cc} \theta_{i}=\theta_{0},\eta_{i}=\eta ; & i=1,2,\ldots r,\\ \theta_{i}=\theta_{0}+\Delta ,\eta_{i}=\eta ; & i\geq r+1\text{,} \end{array}\right.$where *r* ≥ 1 is an unknown time of change, and the values of Δ and *η* are also unknown.

Denote *ψ* = (*θ*, *η*). The log‐likelihood function at the k‐th interim analysis is $I\left(\psi \right)=\sum_{i=1}^{k}\;lnf\left(X_{i},\psi \right)$, and the score vector for *θ* and *η* is defined by

(11) $$V_{k}\left(\theta_{0},\eta \right)=\frac{\partial l\left(\psi \right)}{\partial \psi }=\sum_{i=1}^{k}\;\frac{\partial }{\partial \psi }\text{ln In}f_{\theta_{0}\eta }\left(X_{i}\right)\text{.}$$

In order to define a test statistic for the hypotheses about *θ*, a Fisher information matrix *I* for k observations is partitioned as

$$I=\left(\begin{array}{cc} I_{\theta \theta } & I_{\theta \eta }\\ I_{\eta \theta } & I_{\eta \eta } \end{array}\right)\text{,}$$

where I11=−E∂2∂θ2 Iθη, I22=−E∂2∂η2 Iθη and I12=I21t=−E∂2∂θ∂η Iθη.

Replacing the nuisance parameter *η* with its restricted maximum likelihood estimate ${\hat{\eta }}_{k}$, the conditional efficient score vector *V*_*k*_ is given by

(12) $$V_{k}\left(\theta_{0},{\hat{\eta }}_{k}\right)=\sum_{i=1}^{k}\;\frac{\partial }{\partial \theta }\text{ln}f_{\theta_{0}\hat{\eta }}\left(X_{i}\right)\text{.}$$

This vector is also sometimes termed effective score vector, and its variance $\Gamma_{k}\left(\theta_{0},\eta \right)=I_{11}-I_{12}I_{22}^{-1}I_{21}$ is called effective information, Bera and Bilias (2001). Note that for independent and identically distributed r.v.'s, this variance increases linearly with the number of observations: *Γ*_*k*_(*θ*_0_, *η*) = *kΓ*_1_(*θ*_0_, *η*). Under some standard regularity conditions given in Gombay and Serban (2005), guaranteeing the existence and consistence of a sequence of maximum likelihood estimators, and additionally conditions required by the Law of Iterated Logarithm, Gombay and Serban (2005) showed that under *H*_0_, as *k* → ∞, the effective score vector can be written as

(13) $$V_{k}\left(\theta_{0},{\hat{\eta }}_{k}\right)=\sum_{i=1}^{k}\;Z_{i}+O\left(\text{ln ln(k)}\right)\text{,}$$

where Z_*i*_ are independent identically distributed random variables with expected value E[*Z*_*i*_] = 0 and the covariance matrix cov(*Z*_*i*_) = *k* ^− 1^ *Γ*_*k*_(*θ*_0_, *η*). It follows that the scaled statistic

(14) $$T_{k}=\sqrt{k}\Gamma_{k}{\left(\theta_{0},\eta \right)}^{-1/2}\sum_{i=1}^{k}\;\frac{\partial }{\partial \theta }\text{ln}f_{\theta_{0},{\hat{\eta }}_{k}}\text{,}$$

which is essentially the cumulative sum of deviations from *H*_0_, is asymptotically (*k* → ∞) the cumulative sum of independent identically distributed random variables with mean 0 and variance 1, and thus, a sequence of statistics {*T*_*k*_} can be approximated by a standard Wiener process. In order to use the statistic *T*_*k*_ in practice, the covariance *Γ*_*k*_(*θ*_0_, *η*) is replaced with its estimate $\Gamma_{k}\left(\theta_{0},{\hat{\eta }}_{k}\right)$. Gombay (2003) and Gombay and Serban (2005) introduced a sequential change detection test for Δ > 0 based on the maximum of K‐1 cumulative statistics *T*_*k*_ given by Equation (14) for *k* = 2, …, *K* (or their absolute values, for two‐sided alternatives) as follows. For *k* = 2, 3, …, *K*, where K is a truncation point, reject *H*_0_ in favour of a positive change Δ > 0 at time k if

(15) $$T_{k}\geq \sqrt{K}C\left(\alpha \right)\text{,}$$

and if no such k ≤ *K*, exists, do not reject *H*_0_.

Therefore, the Gombay test is a multiple comparisons procedure, comparing up to K‐1 sequential values of the statistics *T*_*k*_ to the same critical value $\sqrt{K}C\left(\alpha \right)$. The critical values *C*(*α*) of this one‐sided test are calculated as the critical values from the standard normal distribution at 1 − *α*/2 level, *z*_1 − *α*/2_, so that, for instance, *C*(0.05) = 1.96. The two‐sided test is based on |*T*_*k*_|, and its asymptotic (*K* → ∞) critical values *C* ^*^(*α*) are provided in Gombay (2003); Gombay and Serban (2005). In particular, *C* ^*^(0.10) = 1.96, *C* ^*^(0.05) = 2.24, *C* ^*^(0.025) = 2.50 and *C* ^*^(0.01) = 2.80. Gombay (2003) also proposed a similar test based on the maximum (over all *k* ≤ *K*) of *k* ^− 1/2^ *T*_*k*_.

### 3.2 Application of the Gombay test to random effects model

To apply the Gombay test in random effects model of meta‐analysis, consider a sequence of independent studies conducted over time. Each study estimates a treatment effect, *y*_*i*_ for *i* = 1, 2, … with variance $\sigma_{i}^{2}$. Under the null hypothesis, *H*_0_, each effect estimate is normally distributed with the same mean *θ* but different variances: $y_{i}∼N\left(\theta ,{\left(w_{i}^{*}\right)}^{-1}\right)$, where $w_{i}^{*}={\left(\tau^{2}+\sigma_{i}^{2}\right)}^{-1}$ is the weight in random effects model. In the following derivation, the variances $\sigma_{i}^{2}$ are assumed to be known and the only nuisance parameter is *τ* ^2^. The location parameter, *θ*, is the population treatment effect, and it is estimated as weighted mean of the individual effect estimates, ${\hat{\theta }}_{k}=\sum_{i=1}^{k}\;{\hat{w}}_{i}^{*}y_{i}/\sum_{i=1}^{k}\;{\hat{w}}_{i}^{*}$ with estimated weights ${\hat{w}}_{i}^{*}$, *k* = 1, 2, …. Let *θ* = *θ*_0_ be the target value of the effect parameter. As more studies are conducted and results are continually combined, the goal is to determine when the combined effect, ${\hat{\theta }}_{k}$, changes significantly from the target value, *θ*_0_, and stop further studies.

The Gombay test was originally proposed for detection of a sudden shift in the effects as typical in industrial applications. However, it can be used for detection of any monotonic trend in the effects, as its sequential values *T*_*k*_ are essentially the accumulated weighted deviations from the target value *θ*_0_, as can be seen from the Equation (17). This is especially useful in the context of meta‐analysis. The power of the Gombay test depends on the timing and the shape of the trend in effects. This will be discussed in more details in the subsequent sections.

The log‐likelihood function of *y*_*i*_ required to define the Gombay test statistic is given by

(16) $$I\left(y_{i}:\theta ,\tau^{2}\right)=\frac{1}{2}\left\{\text{In}{\hat{w}}_{i}^{*}-{\hat{w}}_{i}^{*}{\left(y_{i}-\theta_{0}\right)}^{2}+C\right\}\text{,}$$

where C is a constant. The efficient score statistic (12) is $V_{k}\left(\theta_{0},{\hat{\tau }}^{2}\right)=\sum_{1}^{k}\;{\hat{w}}_{i}^{*}\left(y_{i}-\theta_{0}\right)$. This familiar statistic is routinely used in meta‐analysis for testing a value of the mean in K studies. Its variance is $\Gamma_{k}=\sum_{1}^{k}\;E\left[{\hat{w}}_{i}^{*}\right]$. In the sequential setting, the Gombay test statistic is based on the maximum of the standardized and scaled by $\sqrt{k}$ score statistics (14) given by

(17) $$T_{k}=\frac{\sqrt{k}\sum_{i=1}^{k}\;{\hat{w}}_{i}^{*}\left(y_{i}-\theta_{0}\right)}{\sqrt{\sum_{i=1}^{k}\;E\left[{\hat{w}}_{i}^{*}\right]}}\text{,}$$

see Web Appendix for derivation. Assuming that the expected value $E\left[{\hat{\tau }}_{i}^{2}\right]=\tau^{2}$ for i = 1, 2, …, K, the expected value of the estimated weights in Equation (17) can be approximated by the first term in their Taylor series expansion, $E\left[{\hat{w}}_{i}^{*}\right]=w_{i}^{*}\left(\tau^{2}\right)$. The between‐study variance component *τ* ^2^ is estimated using the full information available from k studies, ${\hat{\tau }}_{k}$, or from all K studies, ${\hat{\tau }}_{K}^{2}$.

The sequential test using the weights $w_{i}^{*}=w_{i}\left({\hat{\tau }}_{k}^{2}\right)$ and $E\left[{\hat{w}}_{i}^{*}\right]=w_{i}^{*}\left({\hat{\tau }}_{k}^{2}\right)$ in (17) and based on the maximum (over all *k* ≤ *K*) of statistics *T*_*k*_ was proposed by Dogo *et al.* (2015). The *τ* ^2^ was estimated by one of the methods by DerSimonian and Laird (1986); Higgins *et al.* (2011); Mandel and Paule (1970) and the REML. In what follows, the Gombay test statistics based on the four above estimators are denoted by GDL, GH, GMP and GREML, respectively.

However, there is an important limitation in respect to the use of the Gombay test in REM. One of the main assumptions of the Gombay derivation is the identical distribution of the observations *y*_*i*_. This is not satisfied in REM, where the variances of estimated effects differ and the sequence {*T*_*k*_} can be approximated by Wiener process only for very large (in comparison to squared truncation point *K* ^2^) within‐studies sample sizes that make within‐study variances $\sigma_{i}^{2}$ negligible, see Web Appendix A.2 for derivation. Not surprisingly, our simulation work in Dogo *et al.* (2015) showed that the poor approximation of the distribution of the Gombay test statistic by Wiener process resulted in a sequential test with extremely poor control of Type I error. In the next section, we derive a bootstrap‐based test for use with the Gombay test statistic.

## 4 Bootstrap‐based retrospective CUSUM‐type test

The parametric bootstrap is an alternative approach that can be used to obtain an accurate distribution of the test statistic under the null hypothesis without the need to rely on asymptotic theory. In this section, we derive the bootstrap critical values for Gombay test (15) based on statistics *T*_*k*_ (17) with the weights $w_{i}^{*}=w_{i}\left({\hat{\tau }}_{K}^{2}\right)$ and the substitution of $w_{i}\left({\hat{\tau }}_{K}^{2}\right)$ for $E\left[{\hat{w}}_{i}^{*}\right]$. As the knowledge of ${\hat{\tau }}_{K}^{2}$ is required at each step *k* ≤ *K*, this is not a sequential test. This is rather a method allowing retrospective analysis of the sequential combined effects in random‐effects meta‐analysis.

Note that if a change in *θ* does happen at some point *r* + 1 for *r* ≥ 1, this will increase an estimate ${\hat{\tau }}_{K}^{2}$ of the between‐study variance *τ* ^2^ by approximately Δ*p*(1 − *p*), for $p=\sum_{i=r+1}^{K}\;w_{i}/\sum_{i=1}^{K}\;w_{i}<1$. As an illustration, the bias of ${\hat{\tau }}_{DL}^{2}$ in this case is calculated in Web Appendix A.3. This positive bias in ${\hat{\tau }}_{K}^{2}$ does not affect the null distribution of the proposed bootstrap test, as the bootstrap samples are generated from the null distribution with the estimated by ${\hat{\tau }}_{K}^{2}$ between‐study variance. However, the power of the bootstrap test may suffer in the result of this variance inflation. This bias in ${\hat{\tau }}_{K}^{2}$ reaches the maximum at *p* = 1/2, that is, when the shift occurs approximately half‐way and is negligible for large enough *K* > > *r*, as it is of order 1/*K*. We provide further discussion of the effects of this bias on the power of the proposed test in Section 5.1.2.

### 4.1 Bootstrap procedure

Consider the following one‐sided and two‐sided retrospective tests for the existence of a shift. The tests are to be performed after conducting K studies. Because a meta‐analysis requires a minimum of two studies to be conducted, the sequential testing starts with a minimum of two studies and stops as soon as a boundary value is reached or after the K‐th analysis. Define statistics *T*_*k*_, for *k* = 2, …, *K* as

(18) $$T_{k}=\frac{\sum_{i=1}^{k}\;w_{i}\left({\hat{\tau }}_{K}^{2}\right)\left(y_{i}-\theta_{0}\right)}{\sqrt{\sum_{i=1}^{k}\;w_{i}\left({\hat{\tau }}_{K}^{2}\right)}}\text{.}$$

**Test:** For *k* = 2, 3, …, K, reject *H*_0_ if $T_{k}\geq \sqrt{K}C\left(\alpha \right)$ (one‐sided) or $|T_{k}|\geq \sqrt{K}C_{*}\left(\alpha \right)$ (two‐sided) and if no such k, *k* ≤ *K*, exists, do not reject *H*_0_.

The critical values *C*(*α*) and C_*_(*α*) are to be calculated by bootstrap. Let

$$G^{*}=\underset{2\leq k\leq K}{\text{max}}\left\{K^{-1/2}T_{k}\right\}\;\text{and}\;G^{**}=\underset{2\leq k\leq K}{min}\left\{K^{-1/2}T_{k}\right\}\text{.}$$

The calculation of the bootstrap critical values is based on the percentiles of the empirical distribution of *G* ^*^ and *G* ^* *^ calculated from the set of bootstrap samples of the data. The step procedure for the calculation is as follows.

1. From the observed data, calculate the effect estimates *y*_*i*_, the estimated sample variances $S_{i}^{2}$, the study sizes, *n*_*i*_ and other sample statistics as required, for *i* = 1, 2, …, K. Calculate ${\hat{\tau }}_{K}^{2}$ using one of the methods in Section 2.1.
2. Use the values of ${\hat{\tau }}_{K}^{2}$, *θ*_0_, the null value of the effect parameter and other sample statistics as required to generate from appropriate distributions B independent bootstrap samples of the effect estimates $\left\{y_{b_{i}},\;i=1,2,\ldots ,K\right\}$ and corresponding within‐studies variances, $\left\{S_{b_{i}}^{2},\;i=1,2,\ldots ,K\right\}$ for *b* = 1, …, *B*. A standard choice for constructing bootstrap test is to use *B* ≥ 1000.
3. Use the bootstrap values $\left\{\left(y_{b_{i}},\;S_{b_{i}}^{2}\right),\;i=1,2,\ldots ,K\right\}$ to calculate the estimate of *τ* ^2^, ${\hat{\tau }}_{b}^{2}$ for the bth bootstrap sample, *b* = 1, …, *B*, and the corresponding estimated weights in random‐effects model $w_{b_{i}}^{*}={\left({\hat{\tau }}_{b}^{2}+S_{b_{i}}^{2}\right)}^{-1}$.
4. For each bootstrap sample *b* = 1, …, *B*, calculate the sequential statistics
  $$T_{bk}=\sum_{i=1}^{k}\;w_{b_{i}}^{*}\left(y_{b_{i}}-\theta_{0}\right)/\sqrt{\sum_{i=1}^{k}\;w_{b_{i}}^{*}},\;2\leq k\leq K\text{.}$$
5. Find the $G_{b}^{*}$ and $G_{b}^{**}$ statistics as follows:
  (19) $$G_{b}^{*}=max_{2\leq k\leq K}\left\{K^{-1/2}T_{bk}\right\};\;G_{b}^{**}=min_{2\leq k\leq K}\left\{K^{-1/2}T_{bk}\right\}\text{.}$$
6. Order the bootstrap replicates $G_{b}^{*}$ and $G_{b}^{**}$, as $G_{\left(1\right)}^{*}\leq G_{\left(2\right)}^{*}\leq \cdots \leq G_{\left(B\right)}^{*}$ and $G_{\left(1\right)}^{**}\leq G_{\left(2\right)}^{**}\leq \cdots \leq G_{\left(B\right)}^{**}$. For a one‐sided test, the upper critical values are given by the [*B* × (1 − *α*) + 1]^*th*^ element in the sequence of { $G_{\left(i\right)}^{*}$}, while the lower critical values are calculated by the [*B* × *α*]^*th*^ element in the sequence of { $G_{\left(i\right)}^{**}$}. Use *α*/2 instead of *α* for the two‐sided test.

There is no reason to rely on the often assumed approximate normality of various meta‐analytic effect measures, or to assume their constant variances, when using a bootstrap‐based test. Therefore, step 2 of the given bootstrap procedure is effect measure specific. In the following discussion, we provide details for three important examples: sample means, mean differences and log‐odds ratios. These and other popular effect measures such as standardized mean differences and relative risks are available in our R program provided in the Web Appendix.

#### 4.1.1 Sample means

When the effect of interest *y*_*i*_ is the sample mean of the *n*_*i*_ normally distributed observations and its estimated variance $S_{i}^{2}=s_{i}^{2}/n_{i}$ for the sample variance $s_{i}^{2}$, the effects are generated as $y_{b_{i}}∼N\left(\theta_{0},{\hat{\tau }}^{2}+S_{i}^{2}\right)$ and the estimates of the within‐studies variances as $S_{b_{i}}^{2}∼S_{i}^{2}\chi_{\left(n_{i}-1\right)}^{2}/\left(n_{i}-1\right)$, for *i* = 1, …, *K*.

#### 4.1.2 Mean differences

When the effect of interest *y*_*i*_ is the difference of the treatment (T) and the control (C) sample means of normally distributed observations, denote sample variances in the two arms by $s_{\text{iT}}^{2}$ and $s_{iC}^{2}$, with the sample sizes *n*_*iT*_ and *n*_*iC*_, respectively. The variance of the mean difference is $S_{i}^{2}=s_{\text{iT}}^{2}/n_{\text{iT}}+s_{iC}^{2}/n_{iC}$. The effects are generated as $y_{b_{i}}∼N\left(\theta_{0},{\hat{\tau }}^{2}+S_{i}^{2}\right)$ and the within‐arms sample variances are generated as $s_{b_{i}T}^{2}∼s_{\text{iT}}^{2}\chi_{\left(n_{\text{iT}}-1\right)}^{2}/\left(n_{\text{iT}}-1\right)$ and $s_{b_{i}C}^{2}∼s_{iC}^{2}\chi_{\left(n_{iC}-1\right)}^{2}/\left(n_{iC}-1\right)$, for *i* = 1, …, *K*. The within‐studies variances are calculated as $S_{b_{i}}^{2}=s_{b_{i}T}^{2}/n_{\text{iT}}+s_{b_{i}C}^{2}/n_{iC}$.

#### 4.1.3 Log odds ratios

Denote the numbers of events in the control and treatment arms of the studies by *X*_*Ci*_ and *X*_*Ti*_, respectively. Discard the studies with *X*_*Ci*_ + *X*_*Ti*_ = 0 and with *X*_*Ci*_ + *X*_*Ti*_ = *n*_*Ti*_ + *n*_*Ci*_ and adjust the total number of studies K accordingly. Let *a* = 0. When *X*_*Ci*_ = 0 or *X*_*Ci*_ = *n*_*Ci*_, take *a* = 1/2. Estimate probabilities *p*_*Ci*_ = (*X*_*Ci*_ + *a*)/(*n*_*Ci*_ + 2*a*). Generate within‐study parameters $\theta_{b_{i}}∼N\left(\theta_{0},{\hat{\tau }}^{2}\right)$, *i* = 1, …, *K*. Given the values of *p*_*Ci*_ and $\theta_{b_{i}}$, the logits in the treatment groups are $\text{logit}\left(p_{Tb_{i}}\right)=\text{logit}\left(p_{Ci}\right)+\theta_{b_{i}}$. Calculate the probabilities $p_{Tb_{i}}$ and simulate the numbers of the study outcomes $X_{Tb_{i}}$ and $X_{Cb_{i}}$ from the binomial distributions $\text{Binom}\left(n_{\mathrm{Ti}},p_{Tb_{i}}\right)$ and *Binom*(*n*_*Ci*_, *p*_*Ci*_), respectively. Following Gart *et al.* (1985), to obtain unbiased estimators of the log odds ratios and their variances, calculate the log odds ratios as $y_{b_{i}}=\text{log}\left[\left(X_{Tb_{i}}+1/2\right)/\left(n_{Ti}-X_{Tb_{i}}+1/2\right)\right]-\text{log}\left[\left(X_{Cb_{i}}+1/2\right)/\left(n_{Ci}-X_{Cb_{i}}+1/2\right)\right]$ and their variances as $S_{b_{i}}^{2}={\left(X_{Tb_{i}}+1/2\right)}^{-1}+{\left(n_{Ti}-X_{Tb_{i}}+1/2\right)}^{-1}+{\left(X_{Cb_{i}}+1/2\right)}^{-1}+{\left(n_{Cb_{i}}-X_{Cb_{i}}+1/2\right)}^{-1}$ for *i* = 1, …, *K*.

## 5 Simulation study

To evaluate the properties of the bootstrap based test presented in Section 4, a simulation study was conducted. The observed estimates of the treatment effect were generated using the normal distribution, $y_{i}∼N\left(\theta_{0}+\Delta ,\sigma_{i}^{2}+\tau^{2}\right)$. The studies sizes were generated using the normal distribution, $n_{i}∼N\left(n,\frac{n}{4}\right)$ rounded to the nearest integer and truncated on the left at 3; n is the average sample size of the studies. Estimates of sample variances, ${\hat{\sigma }}_{i}^{2}$, were generated using scaled chi‐squared distributions, ${\hat{\sigma }}_{i}^{2}∼\frac{\sigma_{i}^{2}}{\left(n_{i}-1\right)}\chi_{n_{i}-1}^{2}$. This choice ensures that $E\left[{\hat{\sigma }}_{i}^{2}\right]=\sigma_{i}^{2}$. Estimated variances of estimated treatment effects *y*_*i*_ are $S_{i}^{2}={\hat{\sigma }}_{i}^{2}/n_{i}$. The data for each simulated meta‐analysis consisted of a total of K estimates of the observed treatment effects, their estimated variances and corresponding sample sizes $\left\{\left(y_{i},S_{i}^{2},n_{i}\right),\;i=1,\ldots ,K\right\}$. For each data set, we calculated four bootstrap‐based tests using different estimators of *τ* ^2^: DerSimonian and Laird (1986); Higgins *et al.* (2011); Paule and Mandel (1982) and REML (GDL, GH, GMP and GREML, respectively), the penalized *Z*‐test by Lan *et al.* (2003) with *λ* = 2 and SMA based on Lan‐DeMets alpha‐spending function (Lan and DeMets, 1983) and Pocock's boundaries as implemented in program ldbands from the R package Hmisc (Harrell, 2015). Following Wetterslev *et al.* (2009), the OIS for SMA was inflated by an adjustment factor (1 − *I* ^2^)^− 1^ for the *I* ^2^ inconsistency index *I* ^2^ = (*Q* − (*K* − 1))/*Q* (this method is referred to as SMA in the rest of the paper). We used one‐sided tests, and the significance level was fixed at *α* = 0.05. The null value of the effect parameter was taken as *θ*_0_ = 0, and the calculation of each bootstrap critical value was based on *B* = 1000 bootstrap replications. We generated 1000 data sets for each of the 270 combinations of the following variables chosen to represent a realistic range of the parameters values:

$$\begin{array}{l} \sigma^{2}=1,\\ \Delta =\left(0.00,,,0.05,,,0.10,,,0.15,,,0.20\right),\\ n=\left(20,,,50,,,100,,,1000\right),\\ K=\left(20,,,50,,,100\right)\;\text{and}\\ \tau^{2}=\left(0.00,,,0.01,,,0.02,,,0.03,,,0.04,,,0.05\right)\text{.} \end{array}$$

For each scenario, the number of times the test rejects the null hypothesis was recorded.

### 5.1 Results

#### 5.1.1 Type I error

**Figure 1:**
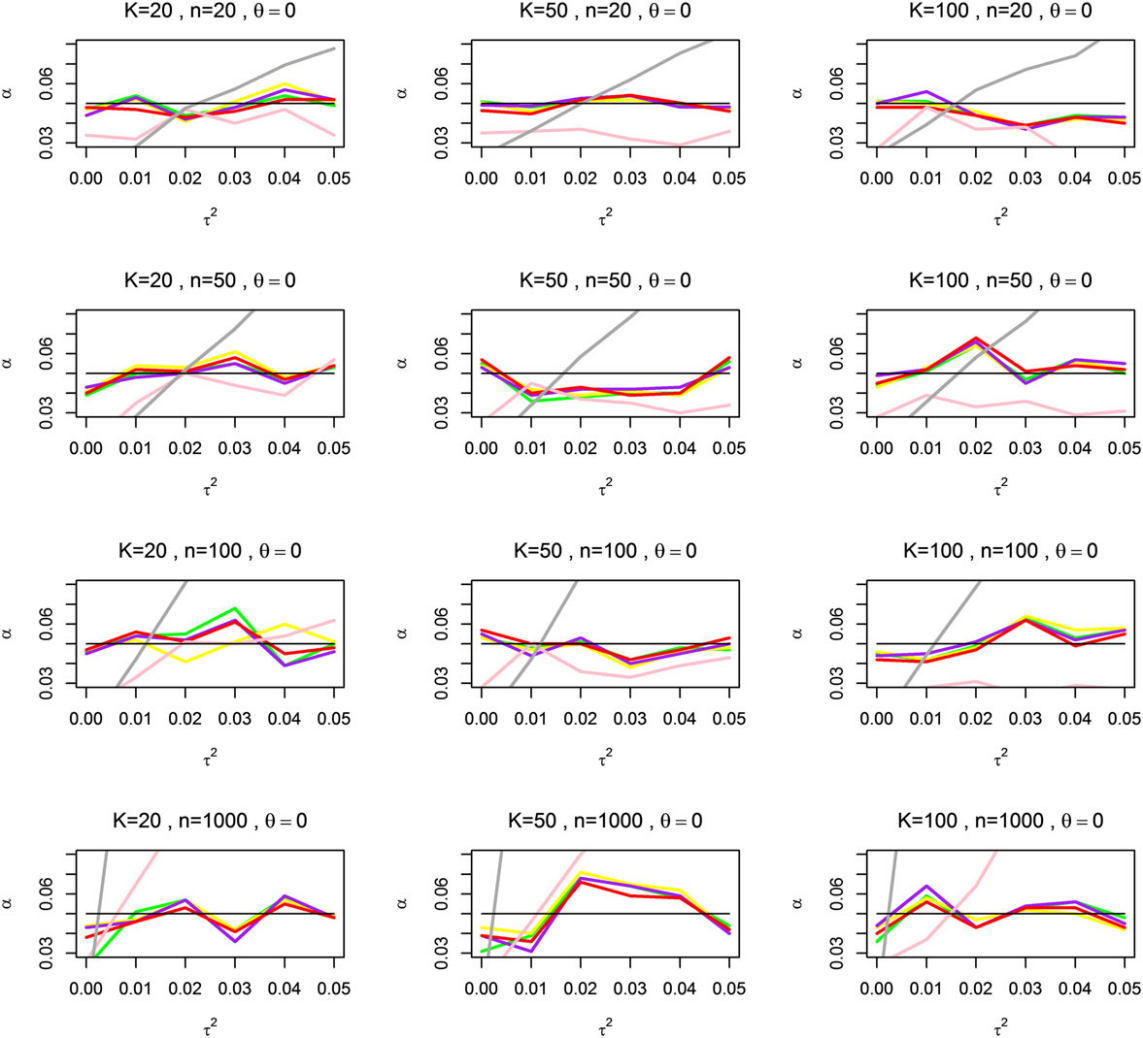
Empirical Type I errors achieved by the bootstrap‐based tests at nominal 5 % level based on DerSimonian and Laird (1986); Higgins *et al.* (2011); Paule and Mandel (1982) and REML estimators of *τ* ^2^ (GDL, GH, GMP and GREML, respectively), the penalized *Z*‐test and SMA. K is the number of studies; n is the average sample size; *Δ* is the effect parameter, *τ* ^2^ is the between‐study variance. The black straight line represents the nominal level of 5% for the test; the yellow, green, purple, red, pink and dark‐grey lines represent GDL, GH, GMP, GREML, penalized *Z*‐test and SMA, respectively. [Colour figure can be viewed at wileyonlinelibrary.com]

Figure 1 compares the overall Type I errors achieved by the bootstrap based tests based on DerSimonian and Laird (1986); Higgins *et al.* (2011); Paule and Mandel (1982) and REML estimators of *τ* ^2^ (GDL, GH, GMP and GREML, respectively), the penalized *Z*‐test and SMA. The Type I errors in bootstrap‐based tests based on all the four estimators of *τ* ^2^ are relatively stable and close to the nominal level. When *K* = 20, the values of Type I errors achieved by GDL and GH are somewhat higher compared with GMP and GREML, but as K increases to 50 and 100, there is very little difference between the four tests, as is clearer from Figure S7 in Web Appendix. Overall, even though there are no clear‐cut winners, it appears that the GMP performs slightly better for smaller studies and the GREML for large studies. In contrast, the Type I errors for the penalized *Z*‐test and the SMA are unsatisfactory. They are far from nominal value of 5 % and increase with increasing values of K, n and *τ* ^2^. Interestingly, the SMA Type I error is mostly below nominal and seems to be stable when *n* ≤ 100 and *K* ≥ 50, but it explodes with increasing *τ* ^2^ when *n* = 1000.

#### 5.1.2 Statistical power

**Figure 2:**
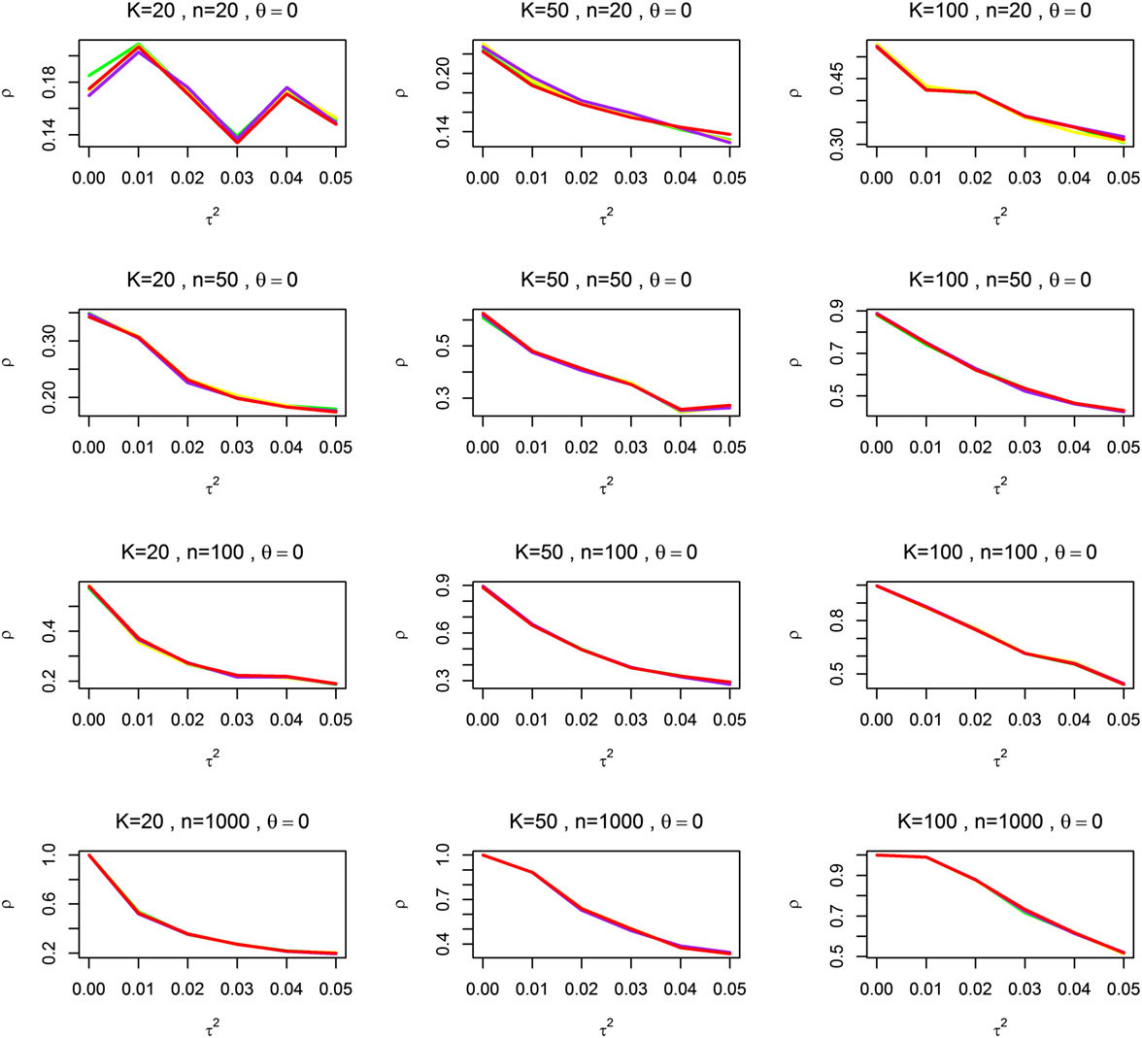
The power of Gombay test for REM with bootstrap critical values based on DerSimonian and Laird (1986); Higgins *et al.* (2011); Paule and Mandel (1982) and REML estimators of *τ* ^2^ (GDL, GH, GMP and GREML) against *θ*. K is the number of studies; n is the average sample size; *ρ* is the power while *Δ* is the effect parameter, *τ* ^2^ is the between‐study variance. The yellow, green, purple and red lines represent GDL, GH, GMP and GREML, respectively. [Colour figure can be viewed at wileyonlinelibrary.com]

Figure 2 and Figures S8–10 in the Web Appendix compare the power of the bootstrap tests based on DerSimonian and Laird (1986); Higgins *et al.* (2011); Paule and Mandel (1982) and the REML estimators of *τ* ^2^ for *r* = 0, that is, when the shift in the mean occurred at the first observation. As expected, for all methods, the power increases with increasing number of studies K, average study size n and value of shift in population treatment effect Δ. However, the power decreases dramatically as *τ* ^2^ increases. This decrease (although not its amount) should be expected as increase in variability makes the detection of an effect more difficult. Comparing power between the four tests in more detail, it is clear that the differences in power are at most 1 % for all values of *τ* ^2^ when Δ = 0.05 (Figure S9 in Web Appendix) and for all values of Δ when *τ* ^2^ = 0.05 (Figure S10 in Web Appendix).

Consider now an alternative scenario of a shift at some point *r* + 1 for 0 < *r* < *K*. This would result in the biased estimation of ${\hat{\tau }}_{K}^{2}$, as discussed in Section 4. Assume, for simplicity, that the within‐study variances are equal. Then the bias in ${\hat{\tau }}_{K}^{2}$ is of order Δ*p*(1 − *p*) for *p* = (*K* − *r*)/*K*, and it reaches maximum for *r* = *K*/2. In this simple case, the weights in REM are also equal and the expected shift in the weighted mean ${\overset{-}{\theta }}_{w}$ is *p*Δ. We can now estimate the change in power. For instance, for Δ = 0.1 and *r* = *K*/2, the equivalent (in respect to power) value of Δ when *r* = 1 is 0.05 and the equivalent *τ* ^2^ value is inflated by 0.01/4 = 0.0025. The latter is a small value, and the power is pretty similar to that given in Figure 2 for Δ = 0.05. Figure S8 in the Web Appendix provides a better understanding of this loss in power, which appears to be almost linear. However, for larger values of Δ, the loss in power will be even greater, as it will be a juxtaposition of the inflation in *τ* ^2^ and the decrease in the equivalent value of Δ. For instance, for Δ = 0.2, the *τ* ^2^ is inflated by 0.01, which has a pronounced effect on power, (cf. Figure 2), whereas the effective value of Δ is decreased to 0.1. For larger values of p, corresponding to longer time elapsed from the shift, the bias in ${\hat{\tau }}^{2}$ is reduced and the power should increase almost linearly with p.

## 6 Examples

To demonstrate the application of the retrospective sequential bootstrap based tests, we consider two examples of medical meta‐analyses. We compare the results of our analysis with the results obtained from CMA, CUSUM, SMA based on Pocock's boundaries and the penalized *Z*‐test. The data for each meta‐analysis were sorted chronologically according to year of publication, from the earliest to the latest. Where the year of publication of two or more studies coincide, the order was selected randomly. Cumulative meta‐analyses were conducted using R package metafor (Viechtbauer, 2010). SMA was based on Lan‐DeMets alpha‐spending function (Lan and DeMets, 1983) and Pocock's boundaries as implemented in program ldbands from the R package Hmisc (Harrell, 2015). CUSUM charts were obtained from the R package *qcc* (Scrucca, 2004).

### 6.1 Magnesium for myocardial infarction

The first application is based on the systematic review conducted by Li *et al.* (2007) to examine the effectiveness of the use of intravenous magnesium for the treatment of acute myocardial infarction. The data consist of 23 trials published from 1984 to 2004, varying in size from 46 to 34 723 patients. The outcome of interest is mortality from acute myocardial infarction and the treatment effects are recorded as log odds ratios. A correction factor 0.5 was added to each entry in the data, and the log odds ratios *ϕ*_*i*_ and their variances $S_{i}^{2}$ were calculated as described in Section 4.1. A negative value of *ϕ*_*i*_ indicates that mortality has been reduced and therefore favours the use of intravenous magnesium. The data and results of the analysis are presented in Tables S1 and S2 of the Web Appendix. A standard random effects meta‐analysis of the data indicates a significant benefit in the use of magnesium with log odds ratio of − 0.2644 (*p*‐value 0.0015), ${\hat{\tau }}_{DL}^{2}=0.037$ and the value of *Q*‐statistic equal to 56.141 with *p*‐value < 0.0001.

To establish the effectiveness of the new intervention, we first test the null hypothesis of no effect of magnesium, that is, *H*_0_: *ϕ* = 0. When the target value is set at 0, the CMA indicates significant effect with the log odds ratio value of − 1.01 (*p*‐value of 0.016) at trial 3. However, this result may be spurious because of the inflated Type I error in CMA. The CUSUM, SMA and the penalized *Z*‐test all indicate a significant effect at trial 7. When the bootstrap tests are used with same target value of 0, the bootstrap critical values for GDL, GH and GMP are all − 0.50 and for GREML, the critical value is − 0.44. GDL and GREML tests reject *H*_0_ at trial 5 and GH and GMP at trial 6, see Figure S11 in Web Appendix B. Hence, for this data, the bootstrap‐based tests are better than the CUSUM, SMA and the penalized Z‐test in terms of early detection.

**Figure 3:**
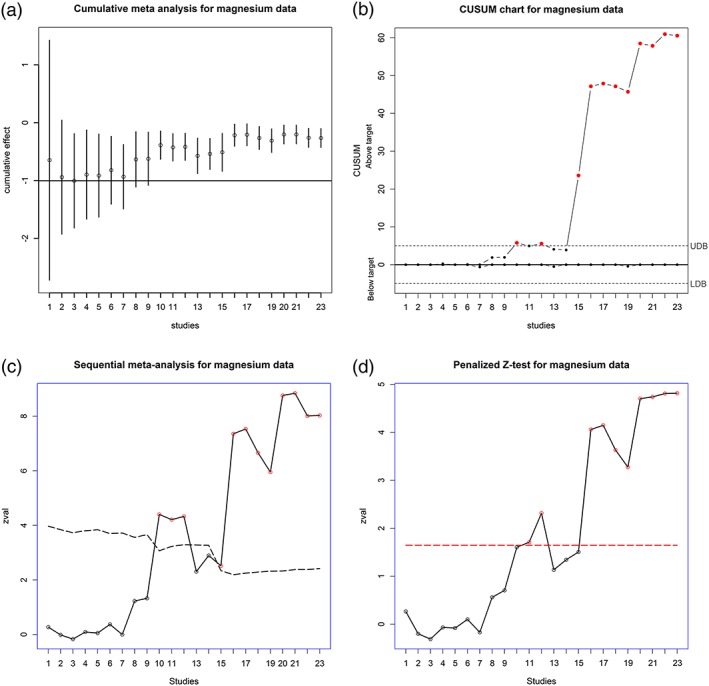
Analysis of magnesium for myocardial infarction (Li *et al.,* 2007) data using cumulative and sequential meta‐analysis, CUSUM and penalized *Z*‐test. CMA and SMA are based on REM and ${\hat{\tau }}_{DL}^{2}$; the horizontal line on CMA plot is the cumulative log odds ratio of − 0.934 (OR of 0.393) at trial 7. The same value of − 0.934 is used as the target value for SMA. The dashed line on the SMA plot is the upper‐boundary value for the one‐sided test, which is first crossed at trial 10. The control limits for CUSUM chart (dashed lines) are defined at ± 5*σ*. The red dashed line on the penalized *Z*‐test plot is the one‐sided upper boundary values. [Colour figure can be viewed at wileyonlinelibrary.com]

**Figure 4:**
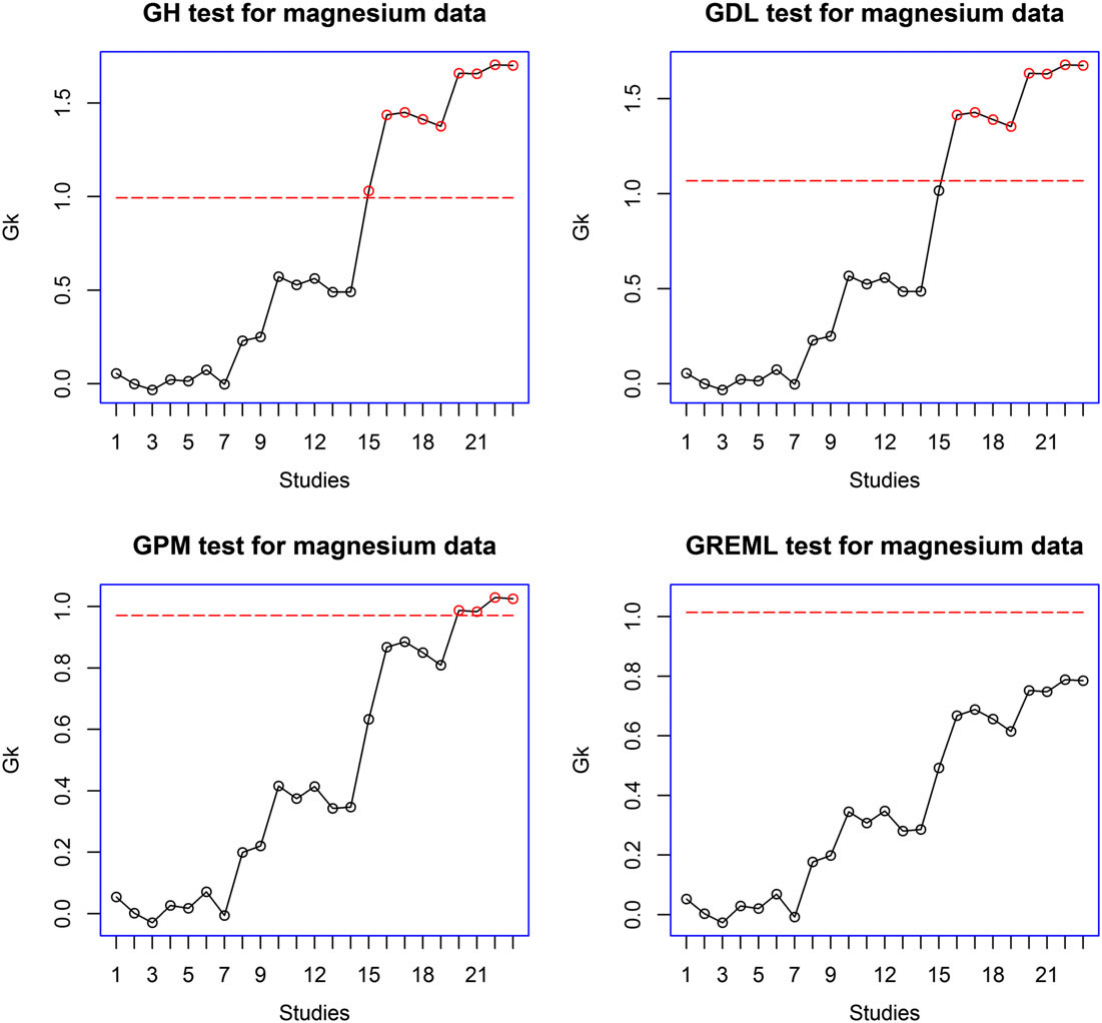
Analysis of magnesium for myocardial infarction (Li *et al.,* 2007) data using bootstrap‐based method based on DerSimonian and Laird (1986); Higgins *et al.* (2011); Paule and Mandel (1982) and the REML estimators of *τ* ^2^ (GDL, GH, GMP and GREML). The target value is set at − 0.934. The red dashed lines are the one‐sided upper boundary values. [Colour figure can be viewed at wileyonlinelibrary.com]

Having established that there is a significant effect of magnesium for acute myocardial infarction, it is important to monitor its effect for any possible trend over time. So we set a new target value of − 0.934 corresponding to the cumulative log odds ratio at trial 7. The CMA plot on Figure 3 exhibits a gradual increase in effect (corresponding to reduction in survival benefit), and the deviation from the horizontal line at − 0.934 becomes significant at trial 10. The CUSUM chart indicates the significant change at trial 10. We expect the CMA and CUSUM to be liberal as they are based on fixed effect boundaries. The SMA with the same target value crosses the upper monitoring boundary at trial 15, while the penalized *Z*‐test (Hu *et al.,* 2007; Lan *et al.,* 2003) hovers at the boundary for trials 13–15, before a definite jump at trial 16. In Figure 4, GDL and GH methods indicate a significant change at trial 15, whereas GMP and GREML indicate a significant change much later, at trial 20 for GMP and at trial 22 for GREML. We believe that trial 15 is the more appropriate point to infer significance of the change. In our data, trials 15 and 16 correspond to two subsets of the large ISIS‐4 trial (1995), which demonstrated lack of effect of magnesium. The performance of the bootstrap based tests is consistent with our conclusion in the simulation study that GDL and GH are more liberal tests compared with GMP and GREML when the number of studies in the analysis is not large.

A clinical significance of the changes can be assessed by comparing effect sizes before and after the change. To this end, we performed standard random‐effects meta‐analyses (using $\tau_{DL}^{2}$) for the three subsets of studies: studies 1 to 7, studies 1 to 14 and studies 15 to 23. The first seven studies provide the odds ratio of 0.393 (0.225, 0.678); the first 14 studies result in the OR of 0.582 (0.444, 0.762)_,_ which is not significantly different. However, for the last nine studies, the OR is 0.916 (0.062, 2.968), indicating no effect of magnesium. The overall OR from all 23 studies is 0.768 (0.652, 0.904), quite a difference from the results in the first seven, or in the first 14 studies.

### 6.2 Nicotine replacement therapy for smoking cessation

The second example is based on the systematic review by Stead *et al.* (2008) testing the effectiveness of nicotine replacement therapy (NRT) for smoking cessation. The data consist of 53 trials published from 1979 to 2005. The outcome of interest is the effect of nicotine containing chewing gum compared with control in aiding smoking cessation. The effect measure used is the log relative risk (logRR). The effect *ϕ*_*i*_ and its variance are estimated by

(20) $${\hat{\phi }}_{i}=\text{log}\left[\frac{\left(x_{T}+1/2\right)\left(n_{C}+1/2\right)}{\left(x_{C}+1/2\right)(n_{T}+1/2)}\right]\;\text{and}\;S_{i}^{2}=\frac{n_{T}-x_{T}}{\left(x_{T}+1/2\right)\left(n_{T}+1/2\right)}+\frac{n_{C}-x_{C}}{\left(x_{C}+1/2\right)\left(n_{C}+1/2\right)}\text{.}$$

A positive value of ${\hat{\phi }}_{i}$ means that NRT is effective for smoking cessation. The data and results of the analysis are presented in Tables 3–7 of the Web Appendix. Random effects meta‐analysis indicates a significant logRR of 0.36 (RR = 1.43), *p*‐value < 0.0001; ${\hat{\tau }}_{DL}^{2}=0.017$ and *Q*‐statistic is 65.77 with *p*‐value of 0.09. Given a small value of estimated between‐study variance and only marginally significant heterogeneity from Cochran's Q test, it is interesting to compare the performance of the retrospective bootstrap‐based tests in comparison with the CUSUM, SMA and the penalized *Z*‐test, which are all based on FEM. For a new intervention, the objective is to test the null hypothesis of no effect of chewing gum *H*_0_ : *ϕ* = 0. When the target value is set at 0, CMA indicates a significant result (*p*‐value 0.031) at trial 3; SMA indicates significant result (*Z*‐value of 3.23 is greater than the upper bound of 2.81) at trial 5. The penalized *Z*‐test based on the adjustment factor of *λ* = 2 indicates significant result (test value of 1.92 is greater than *Z*_1 − 0.05_ = 1.64) at trial 7, while the CUSUM indicates a significant result at trial 5. The bootstrap‐based tests ( GDL, GH, GMP and GREML) all produce a significant result at trial 7, see Figure S12 in Web Appendix. There is not much difference in cumulative logRR between trials 5 and 7: *ϕ* = 0.41 at trial 5, and *ϕ* = 0.42 at trial 7.

**Figure 5:**
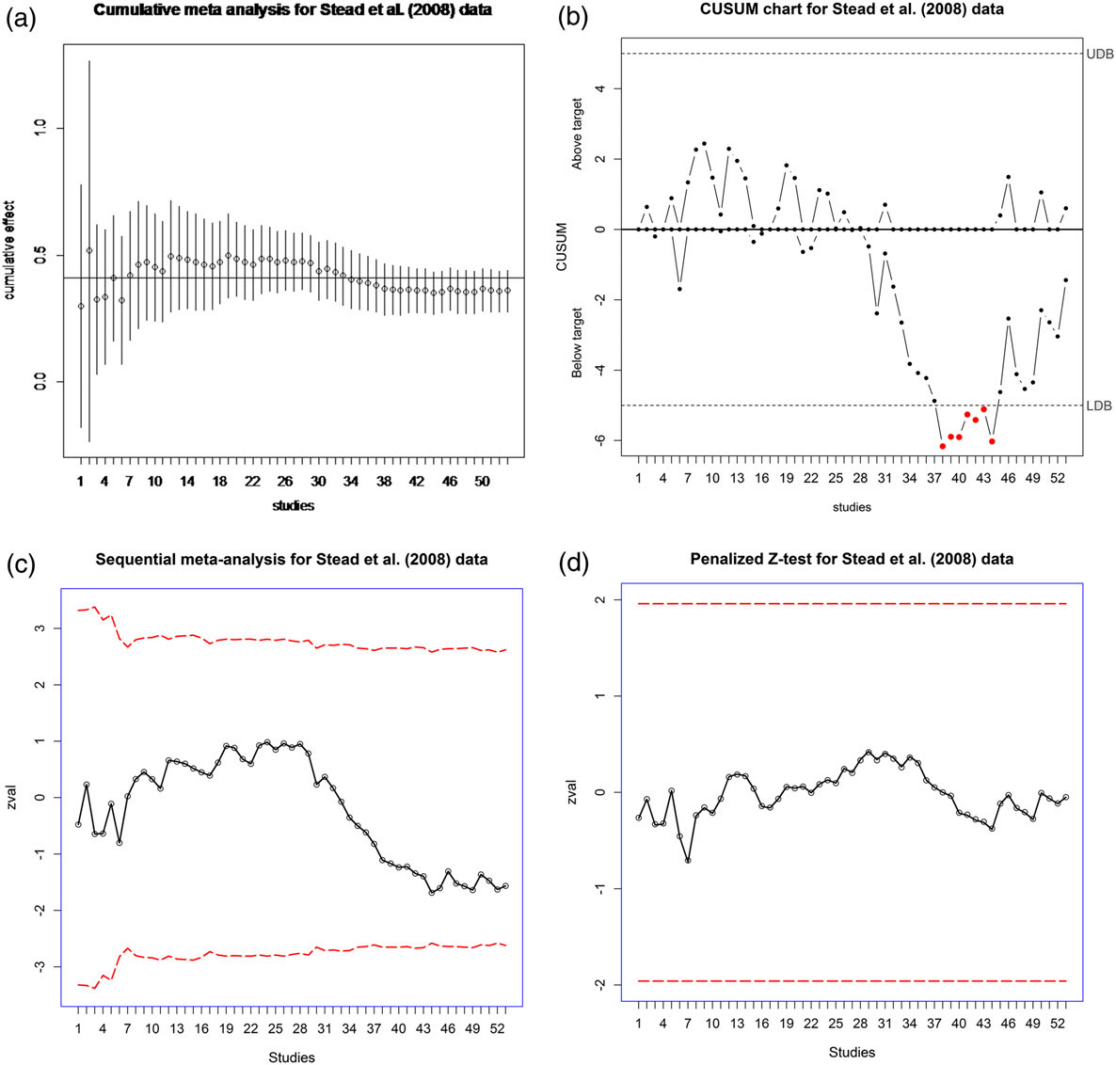
Analysis of Stead *et al.* (2008) data using cumulative and sequential meta‐analysis, CUSUM and penalized *Z*‐test. CMA and SMA are based on REM and ${\hat{\tau }}_{DL}^{2}$; the horizontal line on CMA plot is the cumulative log relative risk of 0.41 (RR of 1.51) at trial 5. The same value of 0.41 is used as the target value for SMA. The control limits for CUSUM chart (dashed lines) are defined at ± 5*σ*. The red solid lines on the SMA plot and the red dashed lines on the penalized *Z*‐test plot are the lower and upper boundary values for two‐sided tests. [Colour figure can be viewed at wileyonlinelibrary.com]

**Figure 6:**
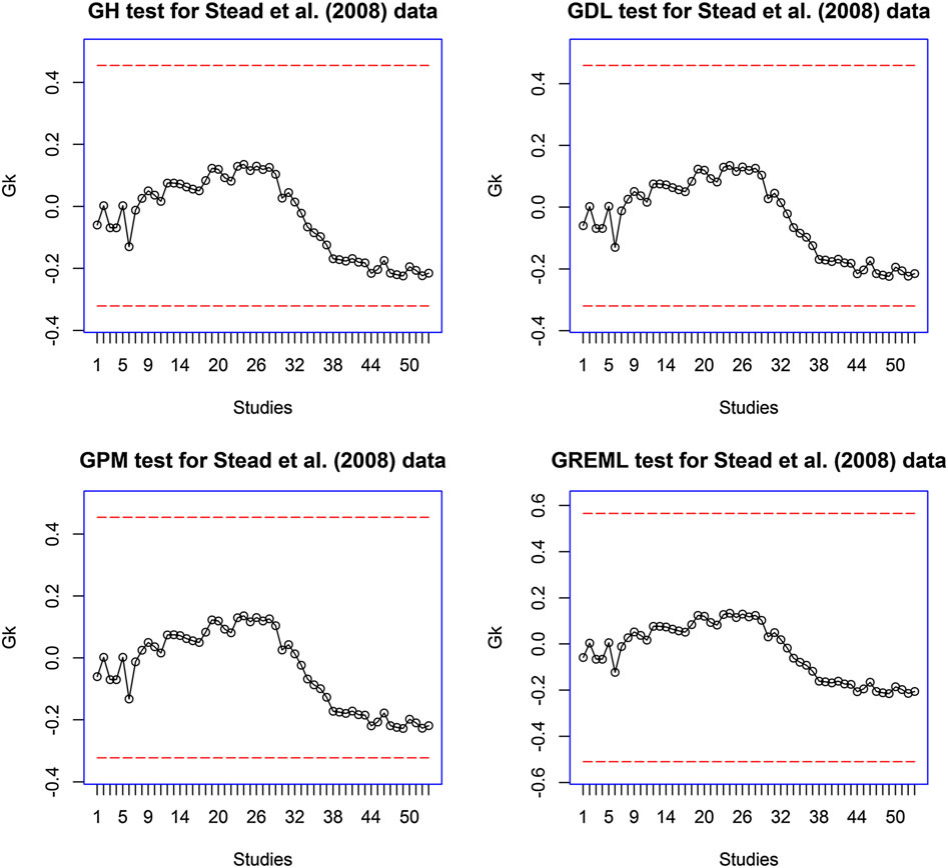
Analysis of Stead *et al.* (2008) data using bootstrap‐based method based on DerSimonian and Laird (1986); Higgins *et al.* (2011); Paule and Mandel (1982) and the REML estimators of *τ* ^2^ (GDL, GH, GMP and GREML). The target value is set at 0.41. The double red dashed lines are the lower and upper boundary values for two‐sided tests. [Colour figure can be viewed at wileyonlinelibrary.com]

To monitor for any further trend in the effect, we set the target value at 0.41 corresponding to the cumulative log relative risk at trial 5 and use two‐sided procedures. As shown in Figures 5 and 6, only the CUSUM indicates a significant result at trial 38. However, the Type I error for the CUSUM is inflated in REM. Overall, we cannot detect any trend in the effects of NRT.

## 7 Summary and conclusion

Temporal changes in the magnitude of the effect sizes reported in many areas of research can be dramatic and lead to the loss or gain of the statistical significance of the cumulative treatment effect, (Kulinskaya and Koricheva, 2010). Numerous sequential methods have been proposed for monitoring the trends in meta‐analysis ( Lau *et al.* (1992); Leimu and Koricheva (2004); Pogue and Yusuf (1997); Wetterslev *et al.* (2008); Higgins *et al.* (2011); Whitehead (1997b); Bollen *et al.* (2006); Kulinskaya and Koricheva (2010); Lan *et al.* (2003)). However, all these methods but Lan *et al.* (2003) are theoretically sound only when monitoring trends in fixed effect model. In this paper, we proposed the use of retrospective CUSUM‐type test based on sequential procedures by Gombay (2003); Gombay and Serban (2005) in combination with bootstrap critical values for sequential random‐effects meta‐analysis. Our simulation results show that the Type I error rates for the new method are closer to the nominal level in comparison to the existing methods and are not affected by increase in the level of heterogeneity *τ* ^2^.

In sequential random‐effects meta‐analysis, the heterogeneity of treatment effect across studies creates inferential problems because of non‐independence of increments. In the proposed method with bootstrap critical values, the problem does not arise as the estimated between‐study variance *τ* ^2^ is included in the calculation of the bootstrap critical values.

Calculation of bootstrap critical values can be computationally intensive. However, with contemporary high performance computers, this should not present much difficulty. Computationally intensive methods involving bootstrapping and permutation tests are becoming common in meta‐analysis (Gumedze and Jackson, 2011). Our R program for calculating the bootstrap‐based CUSUM‐type test with DerSimonian and Laird (1986); Higgins *et al.* (2011); Paule and Mandel (1982) and REML estimators of *τ* ^2^ is provided in the Supporting Information in the online version of this article.

The drawback of using bootstrap‐based critical values is that the resulting method is not a true sequential method and can be used only for retrospective analysis. Even then, it is certainly worthwhile when reviewing the usefulness of an intervention over time. It can be usefully combined with CMA to envisage the trajectory of a cumulative meta‐analysis. Unfortunately, as numerous simulations by us and by other authors have repeatedly demonstrated, well‐behaved sequential methods for random‐effects meta‐analysis are not yet in existence. In contrast, regardless of the method used to estimate *τ* ^2^, the proposed method controls the Type I error irrespective of the number of studies, their sizes and the amount of heterogeneity in treatment effects. We do not have a preferred method of estimating *τ* ^2^ for the test, but we recommend the use of Paule and Mandel (1982) method for smaller studies and the use of the REML for larger studies.

Finally, if and when a change in effect is detected by a sequential test, there is a need to ascertain a practical significance of this change. This can be easily achieved by comparing meta‐effect measures in the original and final meta‐analyses, or before and after the change, as we have done in the Magnesium example in Section 6.1.

## Supporting information

Figure S7: Deviations from nominal 5% level of empirical Type I errors achieved by the bootstrap based tests based on DerSimonian and Laird (1986), Higgins et al. (2011), Paule and Mandel (1982) and REML estimators of *τ* ^2^ (GDL, GH, GMP and GREML, respectively). K is the number of studies; n is the average sample size; △ is the effect parameter, *τ* ^2^ is the between‐study variance. The black straight line is at zero; the yellow, green, purple and red lines corespond to GDL, GH, GMP and GREML, respectively.
Figure S8: The power of Gombay test for REM with bootstrap critical values based on DerSimonian and Laird (1986), Higgins et al. (2011), Paule and Mandel (1982) and REML estimators of *τ* ^2^ (GDL, GH, GMP and GREML) against *Ө*. K is the number of studies; n is the average sample size; *p* is the power while △ is the effect parameter, *τ* ^2^ is the between‐study variance. The yellow, green, purple and the red lines represent GDL, GH, GMP and GREML, respectively.
Figure S9: Comparison of the power of Gombay test for REM with bootstrap critical values based on DerSimonian and Laird (1986), Higgins et al. (2011), Paule and Mandel (1982) and REML estimators of *τ* ^2^ (GDL, GH, GMP and GREML) when Ө = 0:05. K is the number of studies; n is the average sample size; p is the deviations in powers from the average power of the four test while △ is the effect parameter, *τ* ^2^ is the between‐study variance. The black straight line is the nominal value of 5% for the test while the yellow, green, purple and the red lines represent GDL, GH, GMP and GREML, respectively.
Figure S10: Comparison of the power of Gombay test for REM with bootstrap critical values based on DerSimonian and Laird (1986), Higgins et al. (2011), Paule and Mandel (1982) and REML estimators of *τ* ^2^ (GDL, GH, GMP and GREML) when *τ* ^2^ = 0:05. K is the number of studies; n is the average sample size; *p* is the deviations in powers from the average power of the four test while △ is the effect parameter. *τ* ^2^ is the between‐study variance. The black straight line is the nominal value of 5% for the test while the yellow, green, purple and the red lines represent GDL, GH, GMP and GREML, respectively.
Figure S11: Analysis of magnesium data using the bootstrap‐based method based on DerSimonian and Laird (1986), Higgins et al. (2011), Paule and Mandel (1982) and the REML estimators of *τ* ^2^ (GDL, GH, GMP and GREML). The target value is set at 0 and the red dashed lines in GDL, GH, GMP and GREML tests plots are the lower boundary values for one‐sided tests.
Figure S12: Analysis of Stead et al. (2008) data using the bootstrap‐based method based on DerSimonian and Laird (1986), Higgins et al. (2011), Paule and Mandel (1982) and the REML estimators of *τ* ^2^ (GDL, GH, GMP and GREML). The target value is set at 0 and the red dashed lines in GDL, GH, GMP and GREML tests plots are the upper boundary values for one‐sided tests.

Supporting Info Item

Supporting Info Item
