# Supplementary material for: Sequential change detection and monitoring of temporal trends in random‐effects meta‐analysis
Source: Res Synth Methods. 2016 Dec 8;8(2):220–35. doi: 10.1002/jrsm.1222 (PMC5484389; doi:10.1002/jrsm.1222)
Supplement: Supplementary file 1 — Supporting Info Item [file JRSM-8-220-s001.pdf]

# Web Appendix

## A Technical results

### A.1 Proof of equations (16) and (17), Section 3.2

For a normally distributed random variable $y_i \sim (N(\theta, (w_i^*)^{-1})$ , where the weight $w_i^* = (\sigma_i^2 + \tau^2)^{-1}$, log-likelihood is

$$l(y_i : \theta, \tau^2) = \log\left\{(2\pi/w_i^*)^{-1/2}\exp(\frac{w_i^*(y_i-\theta)^2}{2})\right\} = \frac{1}{2}\left\{\log \hat{w}_i^* - \hat{w}_i^*(y_i - \theta_0)^2 + C\right\},$$

as given by (16). The efficient score statistic $V_k(\theta_0, \hat{\tau}^2) = \sum_{i=1}^k \frac{\partial}{\partial\theta}\log f_{\theta_0\hat{\tau}^2}(y_i) = \sum_1^k \hat{w}_i^*(y_i - \theta_0)$. Its variance is $\Gamma_k = \sum_1^k \mathrm{E}[\hat{w}_i^*]$. Substituting these values in equation (14), the Gombay test statistic is given by

$$T_k = \frac{\sqrt{k}\sum\limits_{i=1}^k \hat{w}_i^*(y_i - \theta_0)}{\sqrt{\sum\limits_{i=1}^k \mathrm{E}[\hat{w}_i^*]}}.$$

### A.2 When is the analytical approximation of the sequence $T_k$ by Wiener process valid?, end Section 3.2

Consider a sequence of statistics $T_k$ given by equation (17), for $k \geq 1$. For simplicity, assume known weights $w_i = \sigma_i^2/n_i + \tau^2$ and let the study sample sizes $n_i$ all be of the same order, $n_i = O(n)$. Then the weights $w_i = \tau^{-2}(1 + O(1/n))$, and the sums of weights $W_k = \sum_1^k w_i = \tau^{-2}k(1 + O(1/n))$.

For a sequence $T_k$ to be a Wiener process, we require $\mathrm{E}(T_k) = 0$, $\mathrm{var}(T_k) = k$ (both true, at least approximately), and also independent increments when comparing $T_k$ to $T_{k+u}$, $u \geq 1$. This is equivalent to requiring $\mathrm{cov}(T_k, T_{k+u}) = k$. Let us consider when this condition is approximately true.

$$T_k = \sqrt{\frac{k}{W_k}}[\sum_1^k w_i(y_i - \theta_0)]$$

and

$$T_{k+u} = \sqrt{\frac{k+u}{W_{k+u}}}[\sum_1^k w_i(y_i - \theta_0) + \sum_{k+1}^{k+u} w_i(y_i - \theta_0)] = \sqrt{\frac{(k+u)W_k}{kW_{k+u}}}T_k + \sqrt{\frac{k+u}{W_{k+u}}}\sum_{k+1}^{k+u} w_i(y_i - \theta_0).$$

Therefore $\mathrm{cov}(T_k, T_{k+u}) = \sqrt{\frac{(k+u)W_k}{kW_{k+u}}}k$ and we require $\frac{(k+u)W_k}{kW_{k+u}}k^2 \to k^2$. Since $W_k/k = \tau^{-2}(1 + O(1/n))$, this condition is equivalent to $k^2/n \to 0$ for any $k \leq K$.

This derivation shows that, for the analytically derived test to work, the within-study sample sizes $n$

should be large in comparison to squared truncation point $K^2$. For instance, if $K = 50$, the sample sizes should be large in comparison to 2500, i.e. too large to be practical.

### A.3 Section 4: Illustration of a possible bias in $\hat{\tau}^2_{DL}$ due to a change in location parameter $\theta$

Consider a shift in location $\theta_i = \theta_0$ for $i = 1, \cdots, r$ and $\theta_i = \theta_0 + \delta$ for $i \geq r+1$ for a case of $\tau^2 = 0$. Using the inverse variance weights $w_i = n_i/\sigma_i$ for the $i$th study, the weighted mean $\bar{\theta}_w = \theta_0 + p\Delta$ for $p = \sum_{i=r+1}^{K} w_i / \sum_{i=1}^{K} w_i < 1$. Also $\theta_i - \theta_w = -p\Delta$ for $i \leq r$, and $\theta_i - \theta_w = (1-p)\Delta$ for $i > r$. Therefore, the Cochran's $Q$ statistic is equal to

$$Q = \sum_{i=1}^{r} w_i (p\Delta)^2 + \sum_{i=r+1}^{K} w_i (1-p)^2 \Delta^2 = Wp(1-p)\Delta^2, \text{ for } W = \sum_{1}^{K} w_i.$$

The DerSimonian estimator $\hat{\tau}^2_{DL}$ is obtained as

$$\hat{\tau}^2_{DL} = \frac{Q - (K-1)}{W - \sum w_i^2 / W} = \frac{\Delta^2 p(1-p) - (K-1)/W}{1 - \sum w_i^2 / W^2}.$$

Now let us investigate the order of the terms in the above equation for the case of fixed $r$ and fixed sample sizes $n_i$ and increasing $K$. In the numerator, $p \to 1$ and $1 - p \to 0$ in the limit when $K \to \infty$; also $W = K\bar{w}$ is of order $K$, so $(K-1)/W \to c$ for a constant $c$. $\sum w_i^2/W^2$ is of order $1/K$, so the denominator converges to 1 in the limit $K \to \infty$. Therefore, $\hat{\tau}^2_{DL}$ will take on a negative value, and will be truncated at zero. Thus, for a large enough $K$, an estimated $\hat{\tau}^2_{K}$ is unbiased.

## B Tables

Table 1: Data and results of the meta-analysis of 23 studies on magnesium for myocardial infarction by Li et al. (2009). The subscripts T and C refer to the treatment and control arms of the studies. The columns headed $n_T$ and $n_C$ are the sample sizes, and $x_T$ and $x_C$ are the numbers of events in each study. The columns headed $\varphi$, $v$ and $\varphi_{cum}$ are the log-odds ratios, their variances and the cumulative effects, respectively. The next two columns ($z_{-0.934}$ and $B_{-0.934}$) are the results of SMA; $z_c$ are the values of the Z-statistic for SMA with target value c and $B_c$ are the Pocock's boundaries for the SMA. In the next column ($L_{(-0.934,1.64)}$) are the values of penalized Z-test statistic: $L_{(c,b)}$ are the values of the penalized Z-test statistic with target value c and critical value b. Finally the last eight columns ($GH_{(-0.934,0.61)}$, $GDL_{(-0.934,0.61)}$, $GMP_{(-0.934,0.56)}$, $GREML_{(-0.934,0.54)}$) are the results of Gombay test statistics for REM. $GH_{(c,b)}$, $GDL_{(c,b)}$, $GMP_{(c,b)}$, and $GREML_{(c,b)}$ are the results of Gombay test statistics for REM based on $\hat{\tau^2_H}$, $\hat{\tau^2_{DL}}$, $\hat{\tau^2_{MP}}$ and $\hat{\tau^2_{REML}}$, respectively, c is the target value and b is the bootstrap critical value.

| S/N | Author (Year) | xT | nT | xC | nC | $\hat{\varphi}$ | v | $\varphi_{cum}$ | $z_{-0.93}$ | $B_{-0.93)}$ | $L_{(-0.934,1.6448)}$ |
|---|---|---|---|---|---|---|---|---|---|---|---|
| 1 | Morton (1984) | 1 | 40 | 2 | 36 | -0.65 | 1.12 | -0.65 | 0.2657 | 3.97 | 0.2204 |
| 2 | Rasmussen (1986) | 4 | 56 | 14 | 74 | -1.03 | 0.33 | -0.94 | -0.0045 | 3.84 | 0.0296 |
| 3 | Smith (1986) | 2 | 92 | 7 | 93 | -1.14 | 0.56 | -1.01 | -0.1576 | 3.73 | -0.1025 |
| 4 | Abraham (1987) | 1 | 48 | 1 | 46 | -0.04 | 1.38 | -0.90 | 0.1090 | 3.80 | 0.1296 |
| 5 | Ceremuzynski (1989) | 1 | 25 | 3 | 23 | -1.03 | 1.04 | -0.91 | 0.0679 | 3.84 | 0.0856 |
| 6 | Singh (1990) | 6 | 81 | 11 | 81 | -0.64 | 0.27 | -0.82 | 0.3578 | 3.70 | 0.2596 |
| 7 | Shechter (1990) | 1 | 50 | 9 | 53 | -1.95 | 0.82 | -0.93 | -0.0159 | 3.72 | -0.0336 |
| 8 | Feldsted (1991) | 10 | 150 | 8 | 148 | 0.21 | 0.23 | -0.63 | 1.0997 | 3.56 | 0.6428 |
| 9 | Shechter (1991) | 2 | 21 | 4 | 25 | -0.49 | 0.72 | -0.62 | 1.1996 | 3.66 | 0.7552 |
| 10 | Woods (1992) | 90 | 1150 | 118 | 1150 | -0.30 | 0.02 | -0.39 | 2.7237 | 3.07 | 1.1797 |
| 11 | Wu (1992) | 5 | 125 | 12 | 102 | -1.11 | 0.28 | -0.43 | 2.5141 | 3.23 | 1.0386 |
| 12 | Bhargava (1995) | 3 | 40 | 3 | 38 | -0.06 | 0.63 | -0.42 | 2.6780 | 3.29 | 1.2195 |
| 13 | Shechter (1995) | 4 | 96 | 17 | 98 | -1.49 | 0.30 | -0.57 | 2.3274 | 3.28 | 1.6448 |
| 14 | Thogersen (1995) | 4 | 130 | 8 | 122 | -0.74 | 0.36 | -0.54 | 2.3349 | 3.27 | 1.9990 |
| 15 | ISIS-4a (1995) | 928 | 11675 | 880 | 11648 | 0.06 | 0.002 | -0.51 | 4.8769 | 2.34 | 1.8207 |
| 16 | ISIS-4b (1995) | 1288 | 17333 | 1223 | 17390 | 0.06 | 0.002 | -0.22 | 6.7940 | 2.19 | 4.8188 |
| 17 | Urek (1996) | 1 | 31 | 0 | 30 | 1.10 | 2.73 | -0.21 | 6.8563 | 2.25 | 4.9268 |
| 18 | Raghu (1999) | 6 | 169 | 18 | 181 | -1.05 | 0.22 | -0.26 | 6.6768 | 2.29 | 4.4140 |
| 19 | Gyamlani (2000) | 2 | 50 | 10 | 50 | -1.62 | 0.54 | -0.31 | 6.5017 | 2.32 | 3.9369 |
| 20 | MAGIC (2000) | 475 | 3113 | 472 | 3100 | 0.003 | 0.01 | -0.20 | 7.8455 | 2.33 | 5.6364 |
| 21 | Santoro (2000) | 0 | 75 | 1 | 75 | -1.11 | 2.69 | -0.20 | 7.8282 | 2.38 | 5.7141 |
| 22 | Zhu (2002) | 101 | 1691 | 134 | 1488 | -0.44 | 0.02 | -0.26 | 8.0590 | 2.38 | 5.1986 |
| 23 | Nakashima (2004) | 1 | 89 | 3 | 91 | -0.85 | 0.98 | -0.26 | 8.0384 | 2.48 | 5.1831 |

Table 2: Data and results of the meta-analysis of 23 studies on magnesium for myocardial infarction by Li et al. (2009). (cont'd)

| S/N | $GH_{(0,-0.5016)}$ | $GDL_{(0,-0.4978)}$ | $GMP_{(0,-0.503)}$ | $GREML_{(0,-0.4388)}$ | $GH_{(-0.934,1.01)}$ | $GDL_{(-0.934,1.02)}$ | $GMP_{(-0.93,0.95)}$ | $GREML_{(-0.934,0.78)}$ |
|---|---|---|---|---|---|---|---|---|
| 1 | -0.1256 | -0.1255 | -0.1226 | -0.1199 | 0.0554 | 0.0554 | 0.0541 | 0.0529 |
| 2 | -0.3714 | -0.3709 | -0.3469 | -0.3273 | -0.0010 | -0.0009 | 0.0016 | 0.0037 |
| 3 | -0.4816 | -0.4810 | -0.4538 | -0.4311 | -0.0329 | -0.0329 | -0.0296 | -0.0269 |
| 4 | -0.4550 | -0.4544 | -0.4271 | -0.4043 | 0.0227 | 0.0227 | 0.0295 | 0.0295 |
| 5 | -0.4990 | -0.4985 | -0.4714 | -0.4486 | 0.0141 | 0.0142 | 0.0176 | 0.0204 |
| 6 | -0.5481 | -0.5475 | -0.5155 | -0.4890 | 0.0747 | 0.0746 | 0.0711 | 0.0685 |
| 7 | -0.6608 | -0.6602 | -0.6274 | -0.5998 | -0.0033 | -0.0033 | -0.0063 | -0.0080 |
| 8 | -0.5294 | -0.5290 | -0.5110 | -0.4945 | 0.2299 | 0.2293 | 0.1991 | 0.1770 |
| 9 | -0.5408 | -0.5404 | -0.5217 | -0.5046 | 0.2508 | 0.2501 | 0.2204 | 0.1987 |
| 10 | -0.5620 | -0.5610 | -0.5242 | -0.5024 | 0.5725 | 0.5679 | 0.4151 | 0.3444 |
| 11 | -0.6573 | -0.6563 | -0.6177 | -0.5907 | 0.5288 | 0.5242 | 0.3741 | 0.3067 |
| 12 | -0.6471 | -0.6461 | -0.6049 | -0.5767 | 0.5629 | 0.5584 | 0.4131 | 0.3475 |
| 13 | -0.7656 | -0.7647 | -0.7220 | -0.6882 | 0.4898 | 0.4853 | 0.3420 | 0.2804 |
| 14 | -0.8020 | -0.8011 | -0.7568 | -0.7210 | 0.4913 | 0.4869 | 0.3462 | 0.2856 |
| 15 | -0.6003 | -0.6022 | -0.6386 | -0.6324 | 1.0308 | 1.0169 | 0.6319 | 0.4917 |
| 16 | -0.4780 | -0.4810 | -0.5537 | -0.5645 | 1.4367 | 1.4166 | 0.8657 | 0.6664 |
| 17 | -0.4687 | -0.4716 | -0.5408 | -0.5496 | 1.4496 | 1.4296 | 0.8833 | 0.6867 |
| 18 | -0.5443 | -0.5474 | -0.6178 | -0.6221 | 1.4122 | 1.3922 | 0.8481 | 0.6551 |
| 19 | -0.5974 | -0.6008 | -0.6791 | -0.6852 | 1.3758 | 1.3557 | 0.8071 | 0.6134 |
| 20 | -0.5358 | -0.5393 | -0.6258 | -0.6395 | 1.6594 | 1.6359 | 0.9854 | 0.7506 |
| 21 | -0.5426 | -0.5461 | -0.6342 | -0.6487 | 1.6558 | 1.6323 | 0.9813 | 0.7462 |
| 22 | -0.6471 | -0.6491 | -0.6902 | -0.6867 | 1.7038 | 1.6804 | 1.0272 | 0.7871 |
| 23 | -0.6593 | -0.6614 | -0.7047 | -0.7020 | 1.6996 | 1.6761 | 1.0230 | 0.7832 |

Table 3: Data and results of the meta-analysis of 53 studies on nicotine replacement therapy for smoking cessation by Stead et al. (2008) data. The subscripts T and C refer to the treatment and control arms of the studies. The columns headed $n_T$ and $n_C$ are the sample sizes, and $x_T$ and $x_C$ are the numbers of events in each study. The columns headed $\phi$, $v$ and $\phi_{cum}$ are the log-relative risks, their variances and cumulative effects. The next three columns ($z_{0.41}$, $-B_{0.41}$, $+B_{0.41}$) are the results of SMA; $z_c$ are the value of the z-test for SMA with target value c, $-B_c$ and $+B_c$ the lower and upper Pocock's boundaries for the SMA. The next column ($L_{(-0.934,\pm1.96)}$) provides the results of penalized Z-test; $L_{(c,b)}$ are the values of penalized Z-test with target value c and b is the critical value. Finally the last eight columns are the values of Gombay test statistic for REM. $GH_{(c,b)}$, $GDL_{(c,b)}$, $GMP_{(c,b)}$, and $GREML_{(c,b)}$ are the values of Gombay test statistic for REM based on $\hat{\tau}^2_H$, $\hat{\tau}^2_{DL}$, $\hat{\tau}^2_{MP}$ and $\hat{\tau}^2_{REML}$, respectively, c is the target value and b is the bootstrap critical values or b1 and b2 are the lower and upper critical values, respectively.

| S/N | Author (Year) | xT | nT | xC | nC | $\hat{\phi}$ | v | $\phi_{cum}$ | $z_{0.41}$ | $-B_{0.41}$ | $+B_{0.41}$ | $L_{(0.41,\pm1.96)}$ |
|---|---|---|---|---|---|---|---|---|---|---|---|---|
| 1 | Puska (1979) | 29 | 116 | 21 | 113 | 0.3 | 0.06 | 0.30 | -0.4343 | -3.32 | 3.32 | -0.3895 |
| 2 | Malcom (1980) | 6 | 73 | 3 | 121 | 1.20 | 0.48 | 0.52 | 0.0195 | -3.33 | 3.33 | 0.1889 |
| 3 | Fagerstrom (1982) | 30 | 50 | 23 | 50 | 0.27 | 0.04 | 0.33 | -0.4977 | -3.38 | 3.38 | -0.2360 |
| 4 | Fee (1982) | 23 | 180 | 15 | 172 | 0.38 | 0.10 | 0.34 | -0.4978 | -3.15 | 3.15 | -0.3017 |
| 5 | Jarvis (1982) | 22 | 58 | 9 | 58 | 0.89 | 0.12 | 0.41 | 0.0237 | -3.24 | 3.24 | 0.0786 |
| 6 | Br Thor Society (1983) | 39 | 410 | 111 | 1208 | 0.03 | 0.03 | 0.32 | -0.9410 | -2.82 | 2.82 | -0.6518 |
| 7 | Russell (1983) | 81 | 729 | 78 | 1377 | 0.67 | 0.02 | 0.42 | -0.0843 | -2.67 | 2.67 | 0.0199 |
| 8 | Fegerstrom (1984) | 28 | 96 | 5 | 49 | 1.05 | 0.20 | 0.46 | 0.1909 | -2.80 | 2.80 | 0.2231 |
| 9 | Hjalmarson (1984) | 31 | 106 | 16 | 100 | 0.60 | 0.08 | 0.47 | 0.3709 | -2.83 | 2.83 | 0.3056 |
| 10 | Jamronzik (1984) | 10 | 101 | 8 | 99 | 0.20 | 0.20 | 0.45 | 0.2687 | -2.84 | 2.84 | 0.2172 |
| 11 | Killen (1985) | 16 | 44 | 6 | 20 | 0.19 | 0.16 | 0.44 | 0.1205 | -2.88 | 2.88 | 0.1060 |
| 12 | Clavel (1985) | 24 | 205 | 6 | 222 | 1.47 | 0.20 | 0.50 | 0.5484 | -2.81 | 2.81 | 0.4427 |
| 13 | Hall (1985) | 18 | 41 | 10 | 36 | 0.46 | 0.10 | 0.49 | 0.5521 | -2.86 | 2.86 | 0.4231 |
| 14 | Schneider (1985A) | 9 | 30 | 6 | 30 | 0.41 | 0.21 | 0.48 | 0.5311 | -2.87 | 2.87 | 0.3940 |
| 15 | Schneider (1985B) | 1 | 13 | 3 | 23 | -0.53 | 1.21 | 0.47 | 0.4617 | -2.88 | 2.88 | 0.3375 |
| 16 | Page (1986) | 9 | 93 | 13 | 182 | 0.30 | 0.17 | 0.46 | 0.4114 | -2.83 | 2.83 | 0.2923 |
| 17 | Campbell (1987) | 13 | 424 | 9 | 412 | 0.34 | 0.18 | 0.46 | 0.3681 | -2.73 | 2.73 | 0.2522 |
| 18 | Hall (1987) | 30 | 71 | 14 | 68 | 0.72 | 0.08 | 0.47 | 0.6112 | -2.79 | 2.79 | 0.3981 |
| 19 | Roto (1987) | 19 | 54 | 7 | 60 | 1.10 | 0.16 | 0.0.50 | 0.8982 | -2.81 | 2.81 | 0.5880 |
| 20 | Areechon (1988) | 56 | 99 | 37 | 101 | 0.43 | 0.02 | 0.49 | 0.8750 | -2.80 | 2.80 | 0.5591 |

Table 4: Data and results of the meta-analysis of 53 studies on nicotine replacement therapy for smoking cessation by Stead et al. (2008) data (cont'd)

| S/N | Author (Year) | xT | nT | xC | nC | $\hat{\phi}$ | v | $\phi_{cum}$ | $z_{0.41}$ | $-B_{0.41}$ | $+B_{0.41}$ | $L_{(0.41,\pm1.96)}$ |
|---|---|---|---|---|---|---|---|---|---|---|---|---|
| 21 | Harackiewicz (1988) | 12 | 99 | 7 | 52 | -0.10 | 0.20 | 0.47 | 0.6770 | -2.81 | 2.81 | 0.4336 |
| 22 | Huber (1988) | 13 | 54 | 11 | 60 | 0.27 | 0.13 | 0.46 | 0.5953 | -2.81 | 2.81 | 0.3783 |
| 23 | Llivina (1988) | 61 | 113 | 28 | 103 | 0.69 | 0.03 | 0.48 | 0.9425 | -2.79 | 2.79 | 0.5818 |
| 24 | Tonnesen (1988) | 23 | 60 | 12 | 53 | 0.53 | 0.09 | 0.49 | 0.9854 | -2.81 | 2.81 | 0.6154 |
| 25 | Blondal (1989) | 30 | 92 | 22 | 90 | 0.29 | 0.06 | 0.47 | 0.8434 | -2.79 | 2.79 | 0.5290 |
| 26 | Garcia (1989) | 21 | 68 | 5 | 38 | 0.85 | 0.21 | 0.48 | 0.9498 | -2.81 | 2.81 | 0.5961 |
| 27 | Gilbert (1989) | 11 | 112 | 9 | 111 | 0.19 | 0.18 | 0.47 | 0.8673 | -2.78 | 2.78 | 0.5494 |
| 28 | Huges (1989) | 23 | 210 | 6 | 105 | 0.65 | 0.20 | 0.48 | 0.9158 | -2.76 | 2.76 | 0.5864 |
| 29 | Huges (1990) | 15 | 59 | 5 | 19 | -0.03 | 0.20 | 0.47 | 0.7561 | -2.79 | 2.79 | 0.4837 |
| 30 | Killen (1990) | 129 | 600 | 112 | 617 | 0.17 | 0.01 | 0.44 | 0.2013 | -2.65 | 2.65 | 0.1446 |
| 31 | Nakamura (1990) | 13 | 30 | 5 | 30 | 0.96 | 0.21 | 0.45 | 0.3321 | -2.71 | 2.71 | 0.2259 |
| 32 | Richmond (1990) | 17 | 200 | 14 | 150 | -0.09 | 0.12 | 0.43 | 0.1108 | -2.70 | 2.70 | 0.1055 |
| 33 | Campbell (1991) | 21 | 107 | 21 | 105 | -0.02 | 0.08 | 0.42 | -0.1543 | -2.72 | 2.72 | -0.0601 |
| 34 | Jasen (1991) | 49 | 211 | 19 | 82 | 0.00 | 0.06 | 0.40 | -0.4769 | -2.71 | 2.71 | -0.2890 |
| 35 | Ockene (1991) | 40 | 402 | 33 | 420 | 0.24 | 0.05 | 0.40 | -0.6113 | -2.65 | 2.65 | -0.4078 |
| 36 | Segnan (1991) | 22 | 294 | 37 | 629 | 0.24 | 0.07 | 0.39 | -0.7043 | -2.64 | 2.64 | -0.4995 |
| 37 | Clavel-Chapelon (1992) | 47 | 481 | 42 | 515 | 0.18 | 0.04 | 0.38 | -0.9005 | -2.61 | 2.61 | -0.6720 |
| 38 | Mc Govern (1992) | 51 | 146 | 40 | 127 | 0.10 | 0.03 | 0.37 | -1.2230 | -2.65 | 2.65 | -0.9053 |
| 39 | Mori (1992) | 30 | 178 | 22 | 186 | 0.35 | 0.07 | 0.37 | -1.2443 | -2.65 | 2.65 | -0.9506 |
| 40 | Nebot (1992) | 5 | 106 | 13 | 319 | 0.15 | 0.26 | 0.36 | -1.2774 | -2.65 | 2.65 | -1.0092 |
| 41 | Pirie (1992) | 75 | 206 | 50 | 211 | 0.43 | 0.02 | 0.37 | -1.2200 | -2.64 | 2.64 | -0.9961 |
| 42 | Zelman (1992) | 23 | 58 | 18 | 58 | 0.25 | 0.06 | 0.36 | -1.3056 | -2.67 | 2.67 | -1.1010 |
| 43 | Nieura (1994) | 5 | 84 | 4 | 89 | 0.28 | 0.43 | 0.36 | -1.3196 | -2.66 | 2.66 | -1.1390 |
| 44 | Fortmann (1995) | 110 | 552 | 84 | 522 | 0.21 | 0.02 | 0.35 | -1.5627 | -2.58 | 2.58 | -1.3696 |
| 45 | Gross (1995) | 37 | 131 | 6 | 46 | 0.77 | 0.16 | 0.36 | -1.4740 | -2.63 | 2.63 | -1.2979 |
| 46 | Herrera (1995) | 30 | 76 | 13 | 78 | 0.86 | 0.08 | 0.37 | -1.2630 | -2.64 | 2.64 | -1.0753 |
| 47 | Hall (1996) | 13 | 424 | 9 | 412 | 0.34 | 0.18 | 0.36 | -1.5600 | -2.64 | 2.64 | -1.2521 |

Table 5: Data and results of the meta-analysis of 53 studies on nicotine replacement therapy for smoking cessation by Stead et al. (2008) data (cont'd)

| S/N | Author (Year) | xT | nT | xC | nC | $\hat{\phi}$ | v | $\phi_{cum}$ | $z_{0.41}$ | $-B_{0.41}$ | $+B_{0.41}$ | $L_{(0.41,\pm 1.96)}$ |
|---|---|---|---|---|---|---|---|---|---|---|---|---|
| 48 | Niaura (1999) | 1 | 31 | 2 | 31 | -0.69 | 1.44 | 0.36 | -1.5970 | -1.65 | 1.65 | -1.2826 |
| 49 | Villa (1999) | 11 | 21 | 10 | 26 | 0.31 | 0.10 | 0.35 | -1.6252 | -1.66 | 1.66 | -1.3275 |
| 50 | Garvey (2000) | 75 | 405 | 17 | 203 | 0.79 | 0.06 | 0.37 | -1.4095 | -1.61 | 1.61 | -1.1109 |
| 51 | Cooper (2005) | 17 | 146 | 15 | 147 | 0.13 | 0.11 | 0.36 | -1.4955 | -1.62 | 1.62 | -1.2008 |
| 52 | Ahluwailia (2006) | 53 | 378 | 42 | 377 | 0.23 | 0.04 | 0.36 | -1.6188 | -1.58 | 1.58 | -1.3217 |
| 53 | Moolchan (2008) | 8 | 46 | 2 | 40 | 1.25 | 0.58 | 0.36 | -1.5596 | -1.62 | 1.62 | -1.2734 |

Table 6: Data and results of the meta-analysis of 53 studies on nicotine replacement therapy for smoking cessation by Stead et al. (2008)

data data cont'd

| S/N | $GH_{(0;0.4455)}$ | $GDL_{(0;0.4512)}$ | $GMP_{(0;0.4481)}$ | $GREML_{(0;0.4428)}$ | $GH_{(0.41;-0.32,0.46)}$ | $GDL_{(0.41;-0.33,0.45)}$ | $GMP_{(0.41;-0.31,0.45)}$ | $GREML_{(0.41;-0.32,0.46)}$ |
|---|---|---|---|---|---|---|---|---|
| 1 | 0.1446 | 0.1446 | 0.1456 | 0.1418 | -0.0597 | -0.0597 | -0.0601 | -0.0585 |
| 2 | 0.2242 | 0.2242 | 0.2249 | 0.2223 | 0.0027 | 0.0027 | 0.0021 | 0.0044 |
| 3 | 0.2695 | 0.2695 | 0.2711 | 0.2650 | -0.0684 | -0.0684 | -0.0696 | -0.0647 |
| 4 | 0.3102 | 0.3102 | 0.3119 | 0.3054 | -0.0684 | -0.0684 | -0.0696 | -0.0650 |
| 5 | 0.4132 | 0.4132 | 0.4150 | 0.4079 | 0.0033 | 0.0033 | 0.0022 | 0.0063 |
| 6 | 0.3607 | 0.3607 | 0.3620 | 0.3570 | -0.1293 | -0.1293 | -0.1321 | -0.1213 |
| 7 | 0.5615 | 0.5615 | 0.5668 | 0.5468 | -0.0116 | -0.0116 | -0.0120 | -0.0102 |
| 8 | 0.6130 | 0.6130 | 0.6181 | 0.5989 | 0.0262 | 0.0262 | 0.0256 | 0.0281 |
| 9 | 0.6683 | 0.6683 | 0.6736 | 0.6537 | 0.0509 | 0.0509 | 0.0504 | 0.0525 |
| 10 | 0.6666 | 0.6666 | 0.6719 | 0.6520 | 0.0369 | 0.0369 | 0.0365 | 0.0382 |
| 11 | 0.6622 | 0.6622 | 0.6675 | 0.6475 | 0.0166 | 0.0166 | 0.0162 | 0.0177 |
| 12 | 0.7333 | 0.7333 | 0.7383 | 0.7191 | 0.0753 | 0.0753 | 0.0748 | 0.0770 |
| 13 | 0.7552 | 0.7552 | 0.7603 | 0.7409 | 0.0758 | 0.0758 | 0.0753 | 0.0774 |
| 14 | 0.7635 | 0.7635 | 0.7686 | 0.7492 | 0.0729 | 0.0729 | 0.0725 | 0.0744 |
| 15 | 0.7567 | 0.7567 | 0.7618 | 0.7422 | 0.0634 | 0.0634 | 0.0630 | 0.0647 |
| 16 | 0.7626 | 0.7626 | 0.7678 | 0.7482 | 0.0565 | 0.0565 | 0.0561 | 0.0577 |
| 17 | 0.7685 | 0.7685 | 0.7737 | 0.7541 | 0.0506 | 0.0506 | 0.0502 | 0.0516 |
| 18 | 0.8271 | 0.8271 | 0.8326 | 0.8119 | 0.0840 | 0.0840 | 0.0838 | 0.0845 |
| 19 | 0.8795 | 0.8795 | 0.8849 | 0.8644 | 0.1234 | 0.1234 | 0.1231 | 0.1240 |
| 20 | 0.9304 | 0.9304 | 0.9369 | 0.9124 | 0.1202 | 0.1202 | 0.1200 | 0.1208 |
| 21 | 0.9131 | 0.9131 | 0.9196 | 0.8948 | 0.0930 | 0.0930 | 0.1200 | 0.0933 |
| 22 | 0.9156 | 0.9156 | 0.9222 | 0.8974 | 0.0818 | 0.0818 | 0.0929 | 0.0821 |
| 23 | 1.0038 | 1.0038 | 1.0116 | 0.9820 | 1.1295 | 0.1295 | 0.0817 | 0.1278 |
| 24 | 1.0270 | 1.0276 | 1.0354 | 1.0056 | 1.1354 | 0.1354 | 0.0817 | 0.1336 |
| 25 | 1.0337 | 1.0337 | 1.0416 | 1.0115 | 1.1158 | 0.1158 | 0.1301 | 0.1145 |
| 26 | 1.0569 | 1.0569 | 1.0647 | 1.0349 | 1.1305 | 0.1305 | 0.1360 | 0.1292 |
| 27 | 1.0547 | 1.0547 | 1.0625 | 1.0326 | 1.1191 | 0.1191 | 0.1164 | 0.1178 |
| 28 | 1.0700 | 1.0700 | 1.0778 | 1.0480 | 1.1258 | 0.1258 | 0.1309 | 0.1244 |
| 29 | 1.0568 | 1.0568 | 1.0646 | 1.0347 | 1.1039 | 0.1039 | 0.1196 | 0.1024 |
| 30 | 1.0418 | 1.0418 | 1.0493 | 1.0208 | 1.0277 | 0.0277 | 0.1263 | 0.0321 |

Table 7: Data and results of the meta-analysis of 53 studies on nicotine replacement therapy for smoking cessation by Stead et al. (2008)

data cont'd

| S/N | $GH_{(0;0.4455)}$ | $GDL_{(0;0.4512)}$ | $GMP_{(0;0.4481)}$ | $GREML_{(0;0.4428)}$ | $GH_{(0.41;-0.31,0.45)}$ | $GDL_{(0.41;-0.33,0.45)}$ | $GMP_{(0.41;-0.31,0.45)}$ | $GREML_{(0.41;-0.32,0.46)}$ |
|---|---|---|---|---|---|---|---|---|
| 31 | 1.0674 | 1.0674 | 1.0748 | 1.0467 | 0.0456 | 0.0456 | 0.0438 | 0.0503 |
| 32 | 1.0491 | 1.0491 | 1.0565 | 1.0283 | 0.0152 | 0.0152 | 0.0135 | 0.0197 |
| 33 | 1.0300 | 1.0302 | 1.0375 | 1.0094 | -0.0212 | -0.0212 | -0.0230 | -0.0164 |
| 34 | 1.0079 | 1.0079 | 1.0152 | 0.9875 | -0.0655 | -0.0655 | -0.0676 | -0.0600 |
| 35 | 1.0128 | 1.0128 | 1.0201 | 0.9921 | -0.0840 | -0.0840 | -0.0861 | -0.0781 |
| 36 | 1.0181 | 1.0181 | 1.0255 | 0.9974 | -0.0967 | -0.0967 | =0.0989 | -0.0908 |
| 37 | 1.0174 | 1.0174 | 1.0248 | 0.9966 | -0.1237 | -0.1237 | -0.1261 | -0.1170 |
| 38 | 1.0052 | 1.0052 | 1.0124 | 0.9848 | -0.1682 | -0.1682 | -0.1713 | -0.1598 |
| 39 | 1.0194 | 1.0194 | 1.0268 | 0.9988 | -0.1709 | -0.1709 | -0.1740 | -0.1625 |
| 40 | 1.0201 | 1.0201 | 1.0274 | 0.9994 | -0.1755 | -0.1755 | -0.1785 | -0.1671 |
| 41 | 1.0638 | 1.0638 | 1.0720 | 1.0407 | -0.1676 | -0.1676 | -0.1704 | -0.1598 |
| 42 | 1.0690 | 1.0690 | 1.0772 | 1.0458 | -0.1893 | -0.1793 | -0.1822 | -0.1714 |
| 43 | 1.0703 | 1.0703 | 1.0785 | 1.0471 | -0.1813 | -0.1813 | -0.1841 | -0.1734 |
| 44 | 1.0782 | 1.0782 | 1.0867 | 1.0544 | -0.2147 | -0.2147 | -0.2183 | -0.2047 |
| 45 | 1.0978 | 1.0978 | 1.1063 | 1.0741 | -0.2025 | -0.2025 | -0.2061 | -0.1924 |
| 46 | 1.1397 | 1.1397 | 1.1482 | 1.1157 | -0.1735 | -0.1735 | -0.1771 | -0.1636 |
| 47 | 1.1163 | 1.1163 | 1.1247 | 1.0926 | -0.2143 | -0.2143 | -0.2181 | =0.2038 |
| 48 | 1.1124 | 1.1124 | 1.1208 | 1.0886 | -0.2194 | -0.2194 | -0.2231 | -0.2090 |
| 49 | 1.1191 | 1.1191 | 1.1276 | 1.0953 | -0.2233 | -0.2233 | -0.2271 | -0.2130 |
| 50 | 1.1643 | 1.1643 | 1.1730 | 1.1398 | -0.1936 | -0.1936 | -0.1972 | -0.1838 |
| 51 | 1.1621 | 1.1621 | 1.1708 | 1.1376 | -0.2054 | -0.2054 | -0.2090 | -0.1956 |
| 52 | 1.1682 | 1.1682 | 1.1770 | 1.1434 | -0.2225 | -0.2224 | -0.2261 | -0.2121 |
| 53 | 1.1786 | 1.1786 | 1.1874 | 1.1541 | -0.2142 | -0.2142 | -0.2180 | -0.2038 |

# C Figures

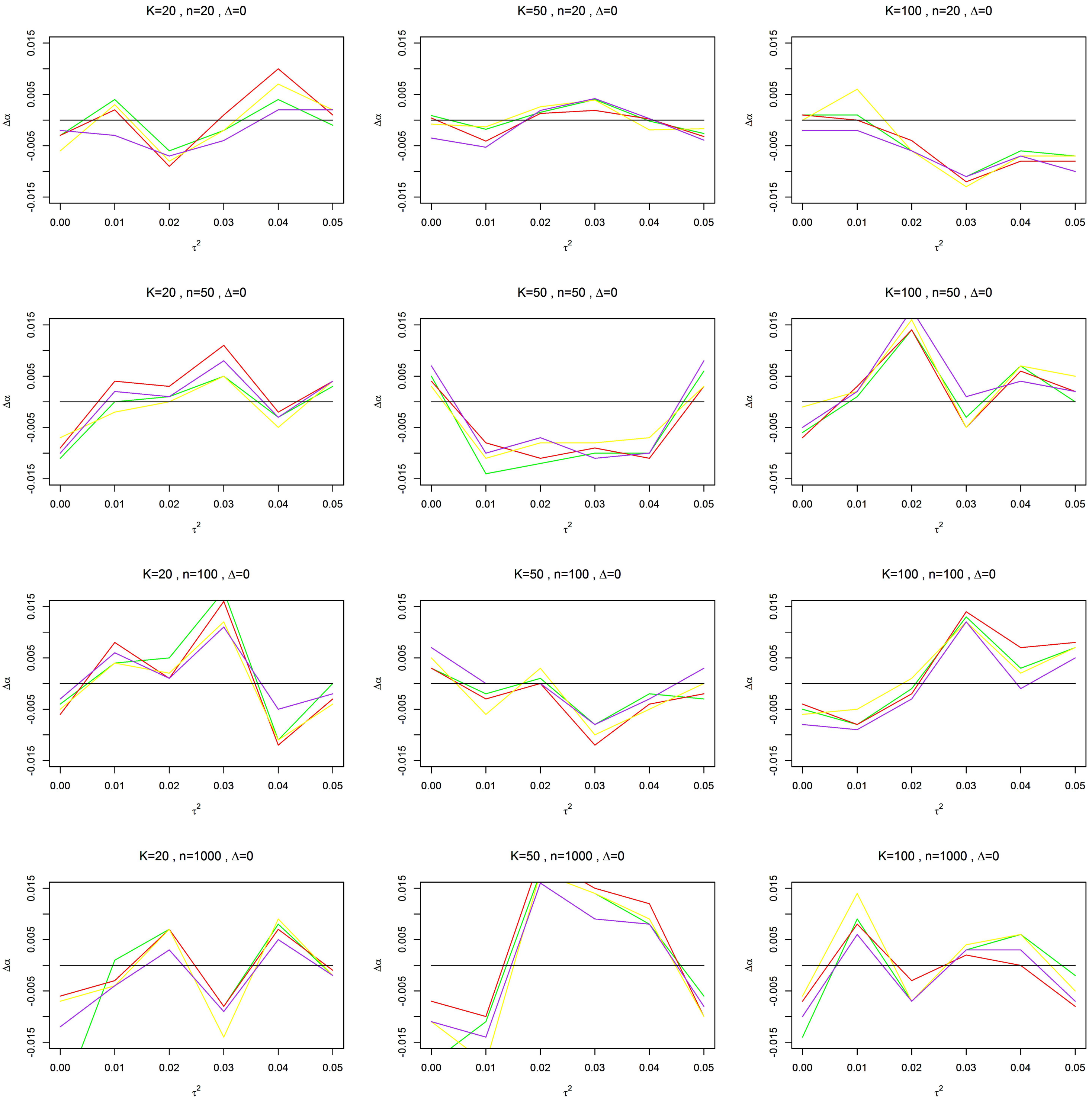


Figure 7: Deviations from nominal 5% level of empirical Type I errors achieved by the bootstrap based tests based on DerSimonian and Laird (1986), Higgins et al. (2011), Paule and Mandel (1982) and REML estimators of $\tau^2$ (GDL, GH, GMP and GREML, respectively). $K$ is the number of studies; $n$ is the average sample size; $\Delta$ is the effect parameter, $\tau^2$ is the between-study variance. The black straight line is at zero; the yellow, green, purple and red lines corespond to GDL, GH, GMP and GREML, respectively.

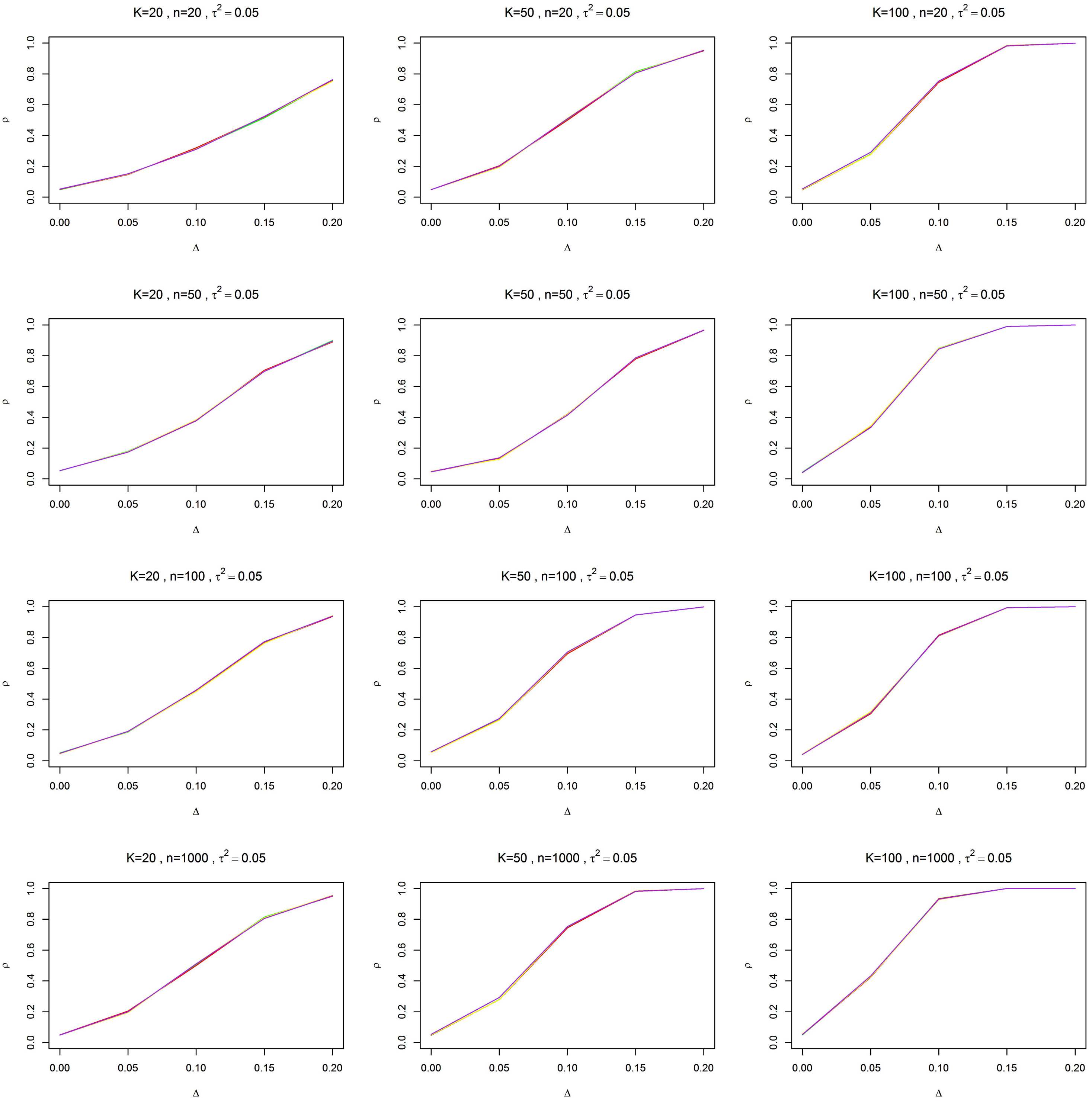


Figure 8: The power of Gombay test for REM with bootstrap critical values based on DerSimonian and Laird (1986), Higgins et al. (2011), Paule and Mandel (1982) and REML estimators of $\tau^2$ (GDL, GH, GMP and GREML) against $\theta$. K is the number of studies; n is the average sample size; $\rho$ is the power while $\Delta$ is the effect parameter, $\tau^2$ is the between-study variance. The yellow, green, purple and the red lines represent GDL, GH, GMP and GREML, respectively.

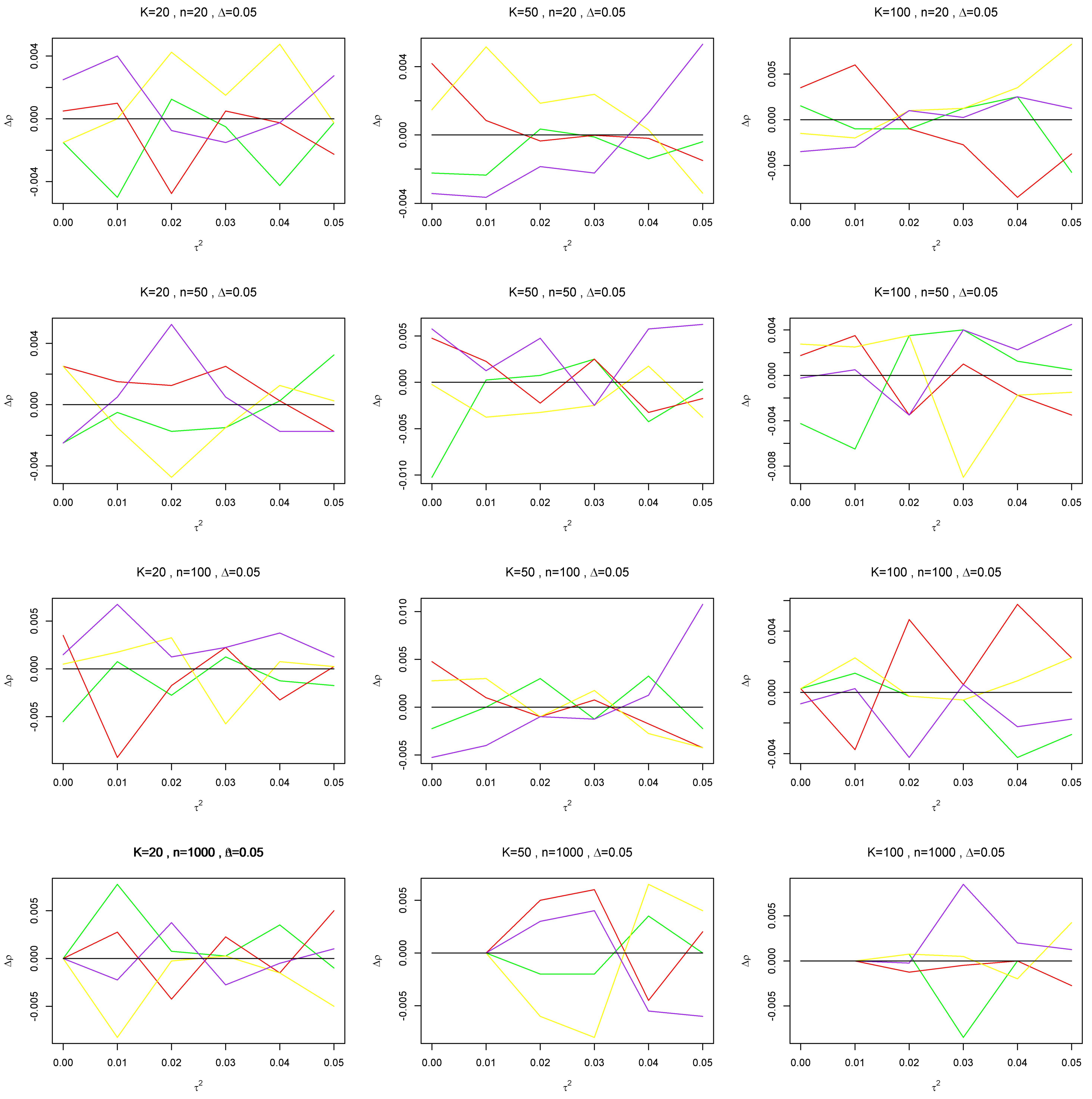


Figure 9: Comparison of the power of Gombay test for REM with bootstrap critical values based on DerSimonian and Laird (1986), Higgins et al. (2011), Paule and Mandel (1982) and REML estimators of $\tau^2$ (GDL, GH, GMP and GREML) when $\theta = 0.05$. K is the number of studies; n is the average sample size; $\rho$ is the deviations in powers from the average power of the four test while $\Delta$ is the effect parameter, $\tau^2$ is the between-study variance. The black straight line is the nominal value of 5% for the test while the yellow, green, purple and the red lines represent GDL, GH, GMP and GREML, respectively.

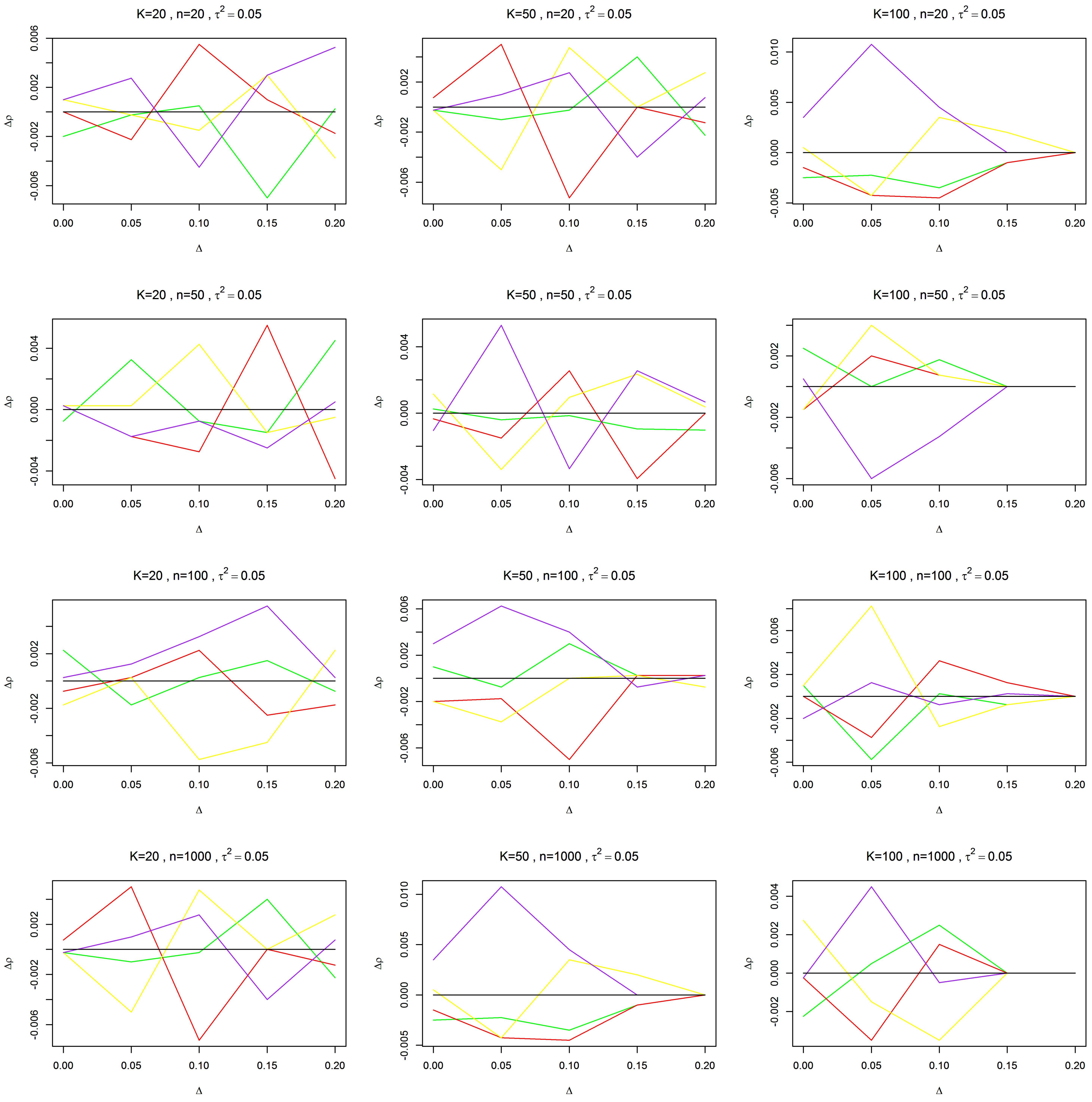


Figure 10: Comparison of the power of Gombay test for REM with bootstrap critical values based on DerSimonian and Laird (1986), Higgins et al. (2011), Paule and Mandel (1982) and REML estimators of $\tau^2$ (GDL, GH, GMP and GREML) when $\tau^2 = 0.05$. K is the number of studies; n is the average sample size; $\rho$ is the deviations in powers from the average power of the four test while $\Delta$ is the effect parameter, $\tau^2$ is the between-study variance. The black straight line is the nominal value of 5% for the test while the yellow, green, purple and the red lines represent GDL, GH, GMP and GREML, respectively.

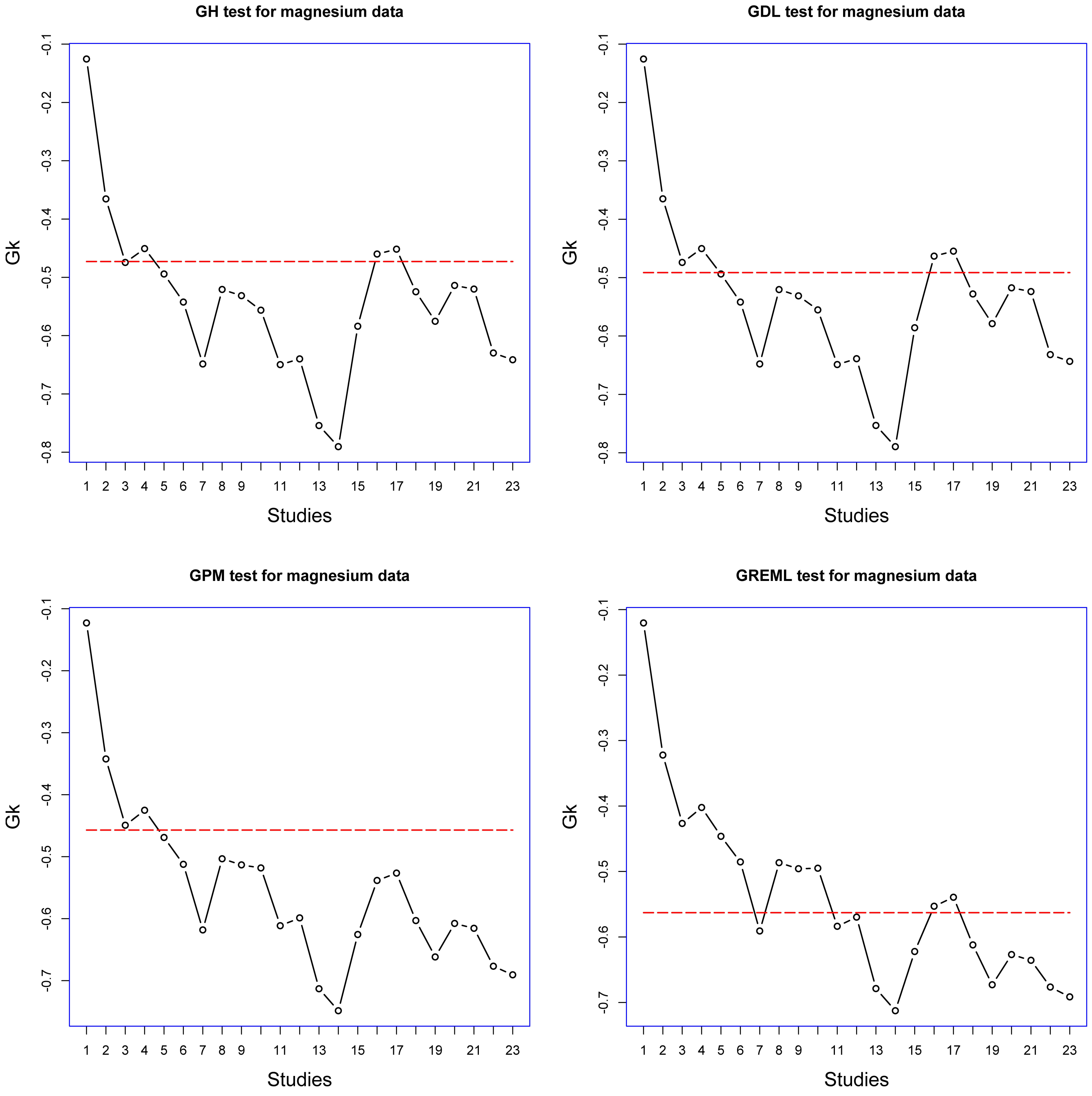


Figure 11: Analysis of magnesium data using the bootstrap-based method based on DerSimonian and Laird (1986), Higgins et al. (2011), Paule and Mandel (1982) and the REML estimators of $\tau^2$ (GDL, GH, GMP and GREML). The target value is set at 0 and the red dashed lines in GDL, GH, GMP and GREML tests plots are the lower boundary values for one-sided tests.

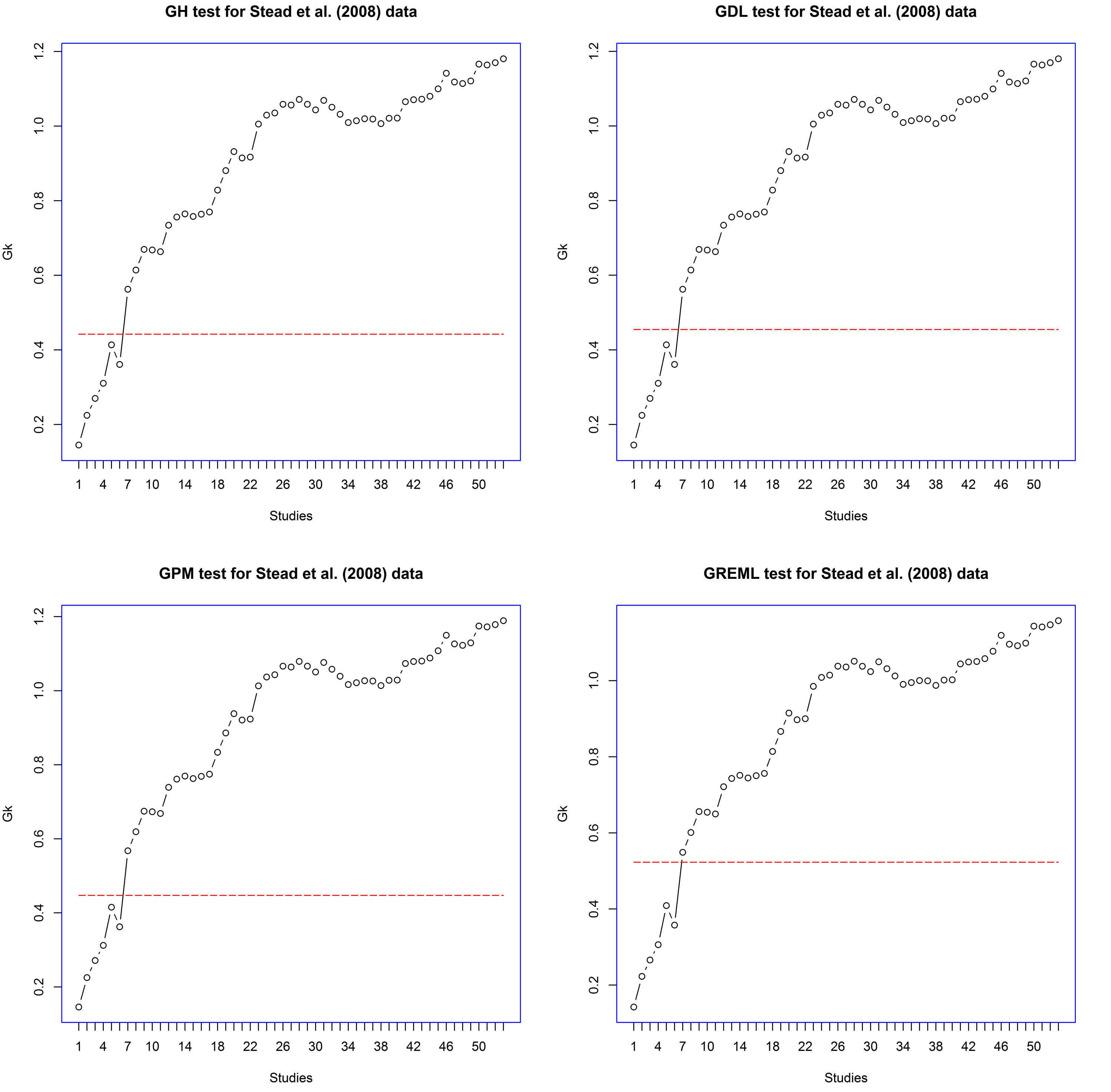


Figure 12: Analysis of Stead et al. (2008) data using the bootstrap-based method based on DerSimonian and Laird (1986), Higgins et al. (2011), Paule and Mandel (1982) and the REML estimators of $\tau^2$ (GDL, GH, GMP and GREML). The target value is set at 0 and the red dashed lines in GDL, GH, GMP and GREML tests plots are the upper boundary values for one-sided tests.

# References


DerSimonian, R. and Laird, N. (1986). Meta-analysis in clinical trials. *Controlled Clinical Trials*, 7(3):177–188.

Higgins, J., Whitehead, A., and Simmonds, M. (2011). Sequential methods for random-effects meta-analysis. *Statistics in Medicine*, 30(9):903–921.

Li, J., Zhang, M., and Egger, M. (2009). Intravenous magnesium for acute myocardial infarction (review). *The Cochrane library.* Issue 1. CD002755.

Paule, R. C. and Mandel, J. (1982). Consensus values and weighting factors. *Journal of Research of the National Bureau of Standards*, 87(5):377–385.

Stead, L., Perera, R.and Bullen, C., Mant, D., and Lancaster, T. (2008). Nicotine replacement therapy for smoking cessation. *Cochrane Database of Systematic Reviews.* Issue 1. CD000146.
