# Supplementary material for: Sequential change detection and monitoring of temporal trends in random‐effects meta‐analysis
Source: Res Synth Methods. 2016 Dec 8;8(2):220–35. doi: 10.1002/jrsm.1222 (PMC5484389; doi:10.1002/jrsm.1222)
Supplement: Supplementary file 2 — Supporting Info Item [file JRSM-8-220-s002.pdf]

Web Appendix

A R programs for calculations in the text

A.1 R programs for calculating $\hat{\tau}^2$

The four (4) programs presented in this section calculate $\hat{\tau}^2$ based on Higgins et al. (2011),DerSimonian and Laird (1986), Paule and Mandel (1982) and REML methods, respectively. The input variables y and v represent the effect estimates and sample variances from studies, respectively.

A.1.1 R program for calculating $\hat{\tau}^2_{DL}$

```
## DESCRIPTION
##############
## This statistic calculate Higgins estimate of tau-squared.
#######################################################################################



## USAGE
## H(y,v).



## ARGUMENTS
##############

## y       A vector of real numbers consisting of effect size estimates from studies.
## v       A vector of non-negative real numbers consisting of sample variances from studies.
#####################################################################################################################

## PROGRAM

H<-function(y,v){
K<-length(y)
if(K<2){
tau<-0
}else{
tau0<-0.01
eta<-1.5
W<-1/v
W2<-W^2
```

```
W3<-W*y
theta<-sum(W3)/sum(W)
Q<-sum(W*(y-theta)^2)
C<-sum(W)-sum(W2)/sum(W)
tauDL<-pmax((Q-(K-1))/(C),0)
tau<-(2*(eta-1)*tau0+(K*tauDL))/(2*(eta-1)+K)
}
tau
}
```

A.1.2 R program for calculating $\hat{\tau}_H^2$

```
## DESCRIPTION
##############
## This statistic calculate DerSimonian-Laired estimate of tau-squared.
######################################################################################


## USAGE
## H(y,v).


## ARGUMENTS
##############

## y       A vector of real numbers consisting of effect size estimates from studies.
## v       A vector of non-negative real numbers consisting of sample variances from studies.
######################################################################################################################

## PROGRAM
DL<-function(y,v){
K<-length(y)
if(K<2){
tau<-0
}else{
W<-1/v
W2<-W^2
W3<-W*y
theta<-sum(W3)/sum(W)
Q<-sum(W*(y-theta)^2)
```

```
C<-sum(W)-sum(W2)/sum(W)
tau<-pmax((Q-(K-1))/(C),0)
}
tau
}
```

A.1.3 R program for calculating $\hat{\tau}^2_{MP}$

```
## DESCRIPTION
##############
## This statistic calculate Paule-Mandel estimate of tau-squared.
######################################################################################


## USAGE
## H(y,v).


## ARGUMENTS
##############

## y       A vector of real numbers consisting of effect size estimates from studies.
## v       A vector of non-negative real numbers consisting of sample variances from studies.
###################################################################################################################

## PROGRAM

MP<-function(y,v){
K<-length(y)
if(K<2){
t<-0
}else{
lb<-0
ub<-1000000000000000
f<-function(t,y,v){
sum((1/(v+t))*(y-sum(y/(v+t))/sum(1/(v+t)))^2)-K+1
}
if(f(lb,y=y,v=v)*f(ub,y=y,v=v)<0){
t<-as.numeric(uniroot(f,c(lb,ub),tol=0.0001,y=y,v=v)[1])
}
else{
```

```
t<-0
}}
t
}
```

A.1.4 R program for calculating $\hat{\tau}^2_{REML}$

```
## DESCRIPTION
##############
## This statistic calculate REML estimate of tau-squared.
####################################################################################



## USAGE
## H(y,v).



## ARGUMENTS
##############

## y       A vector of real numbers consisting of effect size estimates from studies.
## v       A vector of non-negative real numbers consisting of sample variances from studies.
##############################################################################################################

## PROGRAM

RM<-function(y,v){
K<-length(y)
if(K<2){
tau<-0
}else{
tau<-0
M<-0
while(M<K){
tauold<-tau
W<-1/(v+tauold)
W2<-W^2
W3<-W*y
theta<-sum(W3)/sum(W)
W4<-((y-theta)^2)-v
W5<-(W2*W4)
```

```
tau<-pmax((sum(W5)/sum(W2))+1/sum(W),0)
M<-M+1
}
}
tau
}
```

A.2 Program for calculating sequential meta-analysis for REM

```
## DESCRIPTION
##############
## This program works in conjunction with R package ldbounds to calculate the sequential meta-analysis for REM
## based on Pocock's boundary approach and produce graphical analysis for the test.
######################################################################################



## USAGE
## sma(muT,muC,xT,nT,xC,nC,u0,type.E,method,alpha,ty,type.tau)




## ARGUMENTS
##############
## muT:       Sample mean of treatment arm (for continuous data).
## muC:       Sample mean of control arm (for continuous data).
## xT:        Numbers of events of treatment arm (for binary data).
## xC:        Numbers of events of control arm (for binary data).
## vT:        Sample variance from treatment arm.
## vC:        Sample variance from control arm.
## nT:        Sample size of the treatment arm.
## nC:        Sample size of control arm.
## type.E:    Numeric value 1, 2, 3, 4, or 5 that specify the type of effect size to used; 1 is mean diference,
##            2 is standardized mean difference, 3 is odds ratio, 4 is relative risks and 5 is risks difference.
## alpha:     Level of significance of the test.
## u:         Target value
## ty:        Numeric; 1, 2, or 3 that specify the type of test: 1 lower one-sided test, 2 is upper
##            one-sided test and 3 is for two-sided test.
## type.tau:  Numeric 1,2,3 or 4 that specify the method used in estimating tau-squared; 1=Higgins,
##            2=DerSimonian-Laired, 3=Paule-Mandel,4=REML method.
```

```
sma<-function(muT,muC,xT,nT,xC,nC,u0,type.E,method,alpha,ty,type.tau){
if(type.E==1){
y<-muT-muC
v<-vT/nT+vC/nC
}else{
if(type.E==2){
N<-nT+nC
pooledva<r-((nT-1)*vT+(nC-1)*vC)/(N-2)
J<-gamma((N-2)/2)/(sqrt((N-2)/2)*gamma((N-3)/2))
y<-J*(muT-muC)/sqrt(pooledvar)
v<-((N-2)*N*J)/((N-4)*nC*nT)+((N-2)*J^2/(N-4)-1)*y^2
}else{
if(type.E==3){
pT<-(xT+.5)/(nT+.5)
pC<-(xC+.5)/(nC+.5)
y<-log(pT*(1-pC)/(pC*(1-pT)))
v<-1/(xT+.5)+1/(nT-xT)+1/(xC+.5)+1/(nC-xC)
}else{
if(type.E==4){
pT<-(xT+.5)/(nT+.5)
pC<-(xC+.5)/(nC+.5)
y<-log(pT)-log(pC)
v<-1/(xT+.5)-1/(nT+.5)+1/(xC+.5)-1/(nC+.5)
}else{
if(type.E==5){
pT<-(xT+.5)/(nT+.5)
pC<-(xC+.5)/(nC+.5)
y<-pT-pC
v<-(xT+.5)*(nT-xT)/(nT+.5)^3+(xC+.5)*(nC-xC)/(nC+.5)^3
}}}}}


n<-(1/nT+1/nC)^(-1)        ## this statistic calculate the effective sample size.
K<-length(y)
w<-1/v
theta<-sum(w*y)/sum(w)
Q<-sum(w*(y-theta)^2)
```

```
I<-(Q-(K-1))/Q             ## this statistic calculate the rate of inconsistency in treatment effect.
Imax<-sum(n)/(1-I)         ## this statistic calculate the heterogeneity adjusted optimum information size.

est<-IF<-zval<-numeric(K)
for(k in 1:K){
yk<-y[1:k]
vk<-v[1:k]
nk<-n[1:k]
IF[k]<-sum(nk)/Imax      ## this statistic calculate the information fraction at the kth study.
if(type.tau==1){
tauhat<-round(H(y,v),digits=3)
}else{
if(type.tau==2){
tauhat<-round(DL(y,v),digits=3)
}else{
if(type.tau==3){
tauhat<-round(MP(y,v),digits=3)
}else{
if(type.tau==4){
tauhat<-round(RM(y,v),digits=3)
}}}}
wk<-1/(vk+tauhat)
est[k]<-sum(wk*(yk-u0))/sum(wk)
zval[k]<-est[k]/sqrt((sum(wk))^(-1))
}
t<-IF
if((ty==1)|(ty==2)){
alpha<-2*alpha
}else{
alpha<-alpha
}
## This statistic calculate the Pocock's boundary values from the R package ldbounds.
lb<-round(as.numeric(bounds(t=t,iuse=c(2,2),alpha=c(alpha/2,alpha/2))$lower.bound),digits=2)
ub<-round(as.numeric(bounds(t=t,iuse=c(2,2),alpha=c(alpha/2,alpha/2))$upper.bound),digits=2)
if(ty==1){
res<-data.frame(estimates=est,z.value=zval,boundary=lb)
}else{
if(ty==2){
res<-data.frame(estimates=est,z.value=zval,boundary=ub)
}else{
res<-data.frame(estimates=est,z.value=zval,lower.bound=lb,upper.bound=ub)
```

```
}}
## These statistics are for the graphical representation
X<-c(1:K)
if((ty==1)|(ty==2)){
C<-res$boundary
plot(X,zval,axes=FALSE,pch=1,col="black",type="b",ylim=c(min(c(C+.05,zval)),max(c(C+.05,zval))),
xlab=expression(Studies),ylab=expression(paste("zval")),main="Sequential meta-analysis for REM")
par(new=TRUE)
plot(X,C,axes=FALSE,pch=1,lty=5,col="red",type="l",ylim=c(min(c(C+.05,zval)),max(c(C+.05,zval))),
xlab=expression(Studies),ylab=expression(paste("zval")),main="Sequential meta-analysis for REM")
box()
axis(1,at=seq(X[1],max(X),1),labels=TRUE)
axis(2,,labels=TRUE)

}else{
C1<-res$lower.bound
C2<-res$upper.bound
plot(X,zval,axes=FALSE,pch=1,col="black",type="b",ylim=c(min(c(C1-.05,zval)),max(c(C2+.05,zval))),
xlab=expression(Studies),ylab=expression(paste("zval")),main="Sequential meta-analysis for REM")
par(new=TRUE)
plot(X,C1,axes=FALSE,pch=1,col="red",lty=5,type="l",ylim=c(min(c(C1-.05,zval)),max(c(C2+.05,zval))),
xlab=expression(Studies),ylab=expression(paste("zval")),main="Sequential meta-analysis for REM")
par(new=TRUE)
plot(X,C2,axes=FALSE,pch=1,col="red",lty=5,type="l",ylim=c(min(c(C1-.05,zval)),max(c(C2+.05,zval))),
xlab=expression(Studies),ylab=expression(paste("zval")),main="Sequential meta-analysis for REM")
box()
axis(1,at=seq(X[1],max(X),1),labels=TRUE)
axis(2,,labels=TRUE)
}
res
}
```

A.3 Program for calculating the penalised Z-test

```
## DESCRIPTION
##############
## This program calculate the penalised Z-test and produce graphical analysis for the test.
#####################################################################################
```

```
## USAGE
## PZ(muT,muC,xT,nT,xC,nC,u0,type.E,alpha,ty,type.tau,lamda)



## ARGUMENTS
##############
## muT:       Sample mean of treatment arm (for continuous data).
## muC:       Sample mean of control arm (for continuous data).
## xT:        Numbers of events of treatment arm (for binary data).
## xC:        Numbers of events of control arm (for binary data).
## vT:        Sample variance from treatment arm.
## vC:        Sample variance from control arm.
## nT:        Sample size of the treatment arm.
## nC:        Sample size of control arm.
## type.E:    Numeric value 1, 2, 3, 4, or 5 that specify the type of effect size to used; 1 is mean diference,
##            2 is standardized mean difference, 3 is odds ratio, 4 is relative risks and 5 is risks difference.
## alpha:     Level of significance of the test.
## u:         Target value
## ty:        Numeric; 1, 2, or 3 that specify the type of test: 1 lower one-sided test, 2 is upper
##            one-sided test and 3 is for two-sided test.
## type.tau:  Numeric 1,2,3 or 4 that specify the method used in estimating tau-squared; 1=Higgins,
##            2=DerSimonian-Laired, 3=Paule-Mandel,4=REML method.
## lamda:     The adjustment factor for the penalised Z-test.
PZ<-function(muT,muC,xT,nT,xC,nC,u0,type.E,alpha,ty,type.tau,lamda){
if(type.E==1){
y<-muT-muC
v<-vT/nT+vC/nC
}else{
if(type.E==2){
N<-nT+nC
pooledva<r-((nT-1)*vT+(nC-1)*vC)/(N-2)
J<-gamma((N-2)/2)/(sqrt((N-2)/2)*gamma((N-3)/2))
y<-J*(muT-muC)/sqrt(pooledvar)
v<-((N-2)*N*J)/((N-4)*nC*nT)+((N-2)*J^2/(N-4)-1)*y^2
}else{
if(type.E==3){
pT<-(xT+.5)/(nT+.5)
pC<-(xC+.5)/(nC+.5)
y<-log(pT*(1-pC)/(pC*(1-pT)))
```

```
v<-1/(xT+.5)+1/(nT-xT)+1/(xC+.5)+1/(nC-xC)
}else{
if(type.E==4){
pT<-(xT+.5)/(nT+.5)
pC<-(xC+.5)/(nC+.5)
y<-log(pT)-log(pC)
v<-1/(xT+.5)-1/(nT+.5)+1/(xC+.5)-1/(nC+.5)
}else{
if(type.E==5){
pT<-(xT+.5)/(nT+.5)
pC<-(xC+.5)/(nC+.5)
y<-pT-pC
v<-(xT+.5)*(nT-xT)/(nT+.5)^3+(xC+.5)*(nC-xC)/(nC+.5)^3
}}}}}
K<-length(y)
tau<-Z<-P<-Ik<-numeric(K)
for(k in 1:K){
yk<-y[1:k]
vk<-v[1:k]
if(type.tau==1){
tau[k]<-round(H(yk,vk),digits=3)
}else{
if(type.tau==2){
tau[k]<-round(DL(yk,vk),digits=3)
}else{
if(type.tau==3){
tau[k]<-round(MP(yk,vk),digits=3)
}else{
if(type.tau==4){
tau[k]<-round(RM(yk,vk),digits=3)
}}}}

if(tau[k]==0){
tau[k]<-var(vk)
}else{
tau[k]<-tau[k]
}
if(k==1){
wk<-1/vk
}else{
wk<-1/(tau[k]+vk)
```

```
}
Ik<-sum(wk)

Sk<-sum(wk*(yk-u0))
if(Sk<=1){
nn<-1
}else{
nn<-log(log(Ik))
}

P[k]<-pmax(nn,1)

Z[k]<-Sk/sqrt(lamda*Ik*P[k])
}
if((ty==1)|(ty==2)){
alpha<-alpha
}else{
alpha<-alpha/2
}
lb<-rep(qnorm(alpha,lower.tail=TRUE),K)
ub<-rep(qnorm(alpha,lower.tail=FALSE),K)
if(ty==1){
res<-data.frame(z.value=Z,boundary=lb)
}else{
if(ty==2){
res<-data.frame(z.value=Z,boundary=ub)
}else{
res<-data.frame(z.value=Z,lower.bound=lb,upper.bound=ub)
}}
## These statistics are for the graphical representation
X<-c(1:K)
if((ty==1)|(ty==2)){
C<-res$boundary
plot(X,Z,axes=FALSE,pch=1,col="black",type="b",ylim=c(min(c(C+.05,Z)),max(c(C+.05,Z))),
xlab=expression(Studies),ylab=expression(paste("zval")),main="Penalised Z-test")
par(new=TRUE)
plot(X,C,axes=FALSE,pch=1,lty=5,col="red",type="l",ylim=c(min(c(C+.05,Z)),max(c(C+.05,Z))),
xlab=expression(Studies),ylab=expression(paste("zval")),main="Penalised Z-test")
box()
axis(1,at=seq(X[1],max(X),1),labels=TRUE)
axis(2,,labels=TRUE)
```

```
}else{
C1<-res$lower.bound
C2<-res$upper.bound
plot(X,Z,axes=FALSE,pch=1,col="black",type="b",ylim=c(min(c(C1-.05,Z)),max(c(C2+.05,Z))),
xlab=expression(Studies),ylab=expression(paste("zval")),main="Penalised Z-test")
par(new=TRUE)
plot(X,C1,axes=FALSE,pch=1,col="red",lty=5,type="l",ylim=c(min(c(C1-.05,Z)),max(c(C2+.05,Z))),
xlab=expression(Studies),ylab=expression(paste("zval")),main="Penalised Z-test")
par(new=TRUE)
plot(X,C2,axes=FALSE,pch=1,col="red",lty=5,type="l",ylim=c(min(c(C1-.05,Z)),max(c(C2+.05,Z))),
xlab=expression(Studies),ylab=expression(paste("zval")),main="Penalised Z-test")
box()
axis(1,at=seq(X[1],max(X),1),labels=TRUE)
axis(2,,labels=TRUE)
}
res
}
```

A.4 Program for calculating the bootstrap based test

```
## DESCRIPTION
##############
## This program calculate the Gombay test with bootstrap based critical values for REM and produce graphical
## analysis of the test.
#####################################################################################


## USAGE
## Gombay(muT,muC,xT,nT,xC,nC,type.E,alpha,u,ty,type.tau)


## ARGUREMENTS
##############
## muT:       Sample mean of treatment arm (for continuous data).
## muC:       Sample mean of control arm (for continuous data).
## xT:        Numbers of events of treatment arm (for binary data).
## xC:        Numbers of events of control arm (for binary data).
```

```
## vT:        Sample variance from treatment arm.
## vC:        Sample variance from control arm.
## nT:        Sample size of the treatment arm.
## nC:        Sample size of control arm.
## type.E:    Numeric value 1, 2, 3, 4, or 5 that specify the type of effect size to used to be used; 1 is mean dif
##            standardized mean difference, 3 is odds ratio, 4 is relative risks and 5 is risks difference.
## alpha:     Level of significance of the test.
## u:         Target value
## ty:        Numeric; 1, 2, or 3 that specify the type of test: 1 lower one-sided test, 2 is upper
##            one-sided test and 3 is for two-sided test.
## type.tau:  Numeric 1,2,3 or 4 that specify the method used in estimating tau-squared; 1=Higgins,
##            2=DerSimonian-Laired, 3=Paule-Mandel,4=REML method.


Gombay<-function(muT,muC,xT,nT,xC,nC,type.E,alpha,u,ty,type.tau){
if(type.E==1){
y<-muT-muC
v<-vT/nT+vC/nC
}else{
if(type.E==2){
N<-nT+nC
pooledva<r-((nT-1)*vT+(nC-1)*vC)/(N-2)
J<-gamma((N-2)/2)/(sqrt((N-2)/2)*gamma((N-3)/2))
y<-J*(muT-muC)/sqrt(pooledvar)
v<-((N-2)*N*J)/((N-4)*nC*nT)+((N-2)*J^2/(N-4)-1)*y^2
}else{
if(type.E==3){

pT<-(xT+.5)/(nT+.5)
pC<-(xC+.5)/(nC+.5)
y<-log(pT*(1-pC)/(pC*(1-pT)))
v<-1/(xT+.5)+1/(nT-xT)+1/(xC+.5)+1/(nC-xC)
}else{
if(type.E==4){
pT<-(xT+.5)/(nT+.5)
pC<-(xC+.5)/(nC+.5)
y<-log(pT)-log(pC)
v<-1/(xT+.5)-1/(nT+.5)+1/(xC+.5)-1/(nC+.5)
}else{
if(type.E==5){
pT<-(xT+.5)/(nT+.5)
```

```
pC<-(xC+.5)/(nC+.5)
y<-pT-pC
v<-(xT+.5)*(nT-xT)/(nT+.5)^3+(xC+.5)*(nC-xC)/(nC+.5)^3
}}}}}

K<-length(y)
C<-numeric(1)
G<-numeric(K)
## These statistics calculate tau estimate of tau-squared.
if(type.tau==1){
tauhat<-round(H(y,v),digits=3)
}else{
if(type.tau==2){
tauhat<-round(DL(y,v),digits=3)
}else{
if(type.tau==3){
tauhat<-round(MP(y,v),digits=3)
}else{
if(type.tau==4){
tauhat<-round(RM(y,v),digits=3)
}}}}


## The section determine the boostrap critical values.
GGa<-GGb<-numeric(2000)
for (i in 1:2000){
SS<-TT<-numeric(K)
## This statistics generate the bootstrap effect size estimates and the sample variances.
if(type.E==1){
bmuT<-rnorm(K,u,sqrt(tauhat+vT))
bmuC<-rnorm(K,0,sqrt(tauhat+vC))
TT<-bmuT-bmuC
SS<-(vT/((nT-1)))*rchisq(K,nT-1)+(vC/((nC-1)))*rchisq(K,nC-1)
}else{
if(type.E==2){
noncentralpar<-rt(K,N-2,sqrt(nT*nC/N)*y)
y<-rnorm(K,u,sqrt(tauhat))
TT<-J*N/noncentralpar
SS<-((N-2)*N*J)/((N-4)*nC*nT)+((N-2)*J^2/(N-4)-1)*TT^2
}else{
if(type.E==3){
```

```
xbar<-rnorm(K,u,sqrt(tauhat))
pTb<-pC*exp(xbar)/(pC*exp(xbar)+1-pC)
xTb<-rbinom(K,nT,pTb)
xCb<-rbinom(K,nC,pC)
TT<-log((xTb+0.5)/(nT+0.5))-log((xCb+0.5)/(nC+0.5))
SS<-(xTb+0.5)^(-1)-(nT+0.5)^(-1)+(xC+0.5)^(-1)-(nC+0.5)^(-1)
}else{
if(type.E==4){
xbar<-pmin(rnorm(K,u,sqrt(tauhat)),-log(pC))
pTb<-pC*exp(xbar)
xCb<-rbinom(K,nC,pC)
xTb<-rbinom(K,nT,pTb)
pTb<-(xTb+.5)/(nT+.5)
pCb<-(xCb+.5)/(nC+5)
TT<-log(pTb)-log(pCb)
SS<-1/(xTb+.5)-1/(nT+.5)+1/(xCb+.5)-1/(nC+.5)
}else{
if(type.E==5){
xbar<-pmin(pmax(rnorm(K,u,sqrt(tauhat)),-pC),1-pC)
pT<-xbar+pC
xCb<-rbinom(K,nC,pC)
xTb<-rbinom(K,nT,pTb)
TT<-xTb/nT-xCb/nC
a<-SS<-numeric(length(nT))
for (mm in 1:length(nT)){
if((xTb[mm]==0)|(xTb[mm]==nT[mm])|(xCb[mm]==0)|(xCb[mm]==nC[mm])){
a[mm]<-.5
}else{
a[mm]<-0
}
SS[mm]<-(xTb[mm]+a[mm])*(nT[mm]-xTb[mm]+a[mm])/(nT[mm]+2*a[mm])^3+(xCb[mm]+a[mm])*(nC[mm]-xCb[mm]+a[mm])/(nC[mm]+2*
}
}}}}}

## These statistics re-calculate tau-squared estimate from the bootstrap data.

if(type.tau==1){
that<-round(H(TT,SS),digits=3)
}else{
if(type.tau==2){
that<-round(DL(TT,SS),digits=3)
```

```
}else{
if(type.tau==3){
that<-round(MP(TT,SS),digits=3)
}else{
if(type.tau==4){
that<-round(RM(TT,SS),digits=3)
}}}}


g<-numeric(K)
for(k in 1:K){


Tk<-SSk<-W<-numeric(k)

Tk<-TT[1:k]

SSk<-SS[1:k]

W<-1/(SSk+that)

#This statistics calculate the Gombay test statistic from bootstrap data.
g[k]<-round(sum(W*(Tk-u))/sqrt(K*sum(W)),digits=4)
}
GGa[i]<-min(g[1:K])
GGb[i]<-max(g[1:K])
}

##This statistics compute the bootstrap critical values based on 4 different estimators of tau.
if(ty==1){    ##lower one-sided critical values
C<- sort(GGa,decreasing=FALSE)[round(length(GGa)*alpha)]
}else{
if(ty==2){   ##upper one-sided critical values
C<- sort(GGb,decreasing=FALSE)[round(length(GGb)*(1-alpha))]
}else{    ##two-sided critical values
C<- c(sort(GGa,decreasing=FALSE)[round(length(GGa)*(alpha/2))],sort(GGb,decreasing=FALSE)
[round(length(GGb)*(1-alpha/2))])
}
}

gg<-numeric(K)
```

```
## This statistic calculate Gombay test statistic for REM based on the real data.

for(k in 1:K){
wk<-Tk<-Sk<-numeric(k)
Tk<-y[1:k]
Sk<-v[1:k]
wk<-1/(Sk+tauhat)
gg[k]<-round(sum(wk*(Tk-u))/sqrt(K*sum(wk)),digits=4)
}

## These statistics are for the graphical representation
X<-c(1:K)
if((ty==1)|(ty==2)){
C<-c(rep(C,K))
results<-data.frame("G"=gg,"bound.G"=C)

plot(X,gg,axes=FALSE,pch=1,col="black",type="b",ylim=c(min(c(C+.05,gg)),max(c(C+.05,gg))),
xlab=expression(Studies),ylab=expression(paste("Gk")),main="Gombay test for REM")
par(new=TRUE)
plot(X,C,axes=FALSE,pch=1,lty=5,col="red",type="l",ylim=c(min(c(C+.05,gg)),max(c(C+.05,gg))),
xlab=expression(Studies),ylab=expression(paste("Gk")),main="Gombay test for REM" )
box()
axis(1,at=seq(X[1],max(X),1),labels=TRUE)
axis(2,,labels=TRUE)

}else{
C1<-c(rep(C[1],K))
C2<-c(rep(C[2],K))
results<-data.frame("G"=gg,"lower.bound"=C1,"upper.bound"=C2)
plot(X,gg,axes=FALSE,pch=1,col="black",type="b",ylim=c(min(c(C1-.05,gg)),max(c(C2+.05,gg))),
xlab=expression(Studies),ylab=expression(paste("Gk")),main="Gombay test for REM")
par(new=TRUE)
plot(X,C1,axes=FALSE,pch=1,col="red",lty=5,type="l",ylim=c(min(c(C1-.05,gg)),max(c(C2+.05,gg))),
xlab=expression(Studies),ylab=expression(paste("Gk")),main="Gombay test for REM")
par(new=TRUE)
plot(X,C2,axes=FALSE,pch=1,col="red",lty=5,type="l",ylim=c(min(c(C1-.05,gg)),max(c(C2+.05,gg))),
xlab=expression(Studies),ylab=expression(paste("Gk")),main="Gombay test for REM")
box()
axis(1,at=seq(X[1],max(X),1),labels=TRUE)
axis(2,,labels=TRUE)
```

```
}
return(results)
}
```

References

DerSimonian, R. and Laird, N. (1986). Meta-analysis in clinical trials. *Controlled clinical trials*, 7(3):177–188.

Higgins, J., Whitehead, A., and Simmonds, M. (2011). Sequential methods for random-effects meta-analysis. *Statistics in medicine*, 30(9):903–921.

Paule, R. C. and Mandel, J. (1982). Consensus values and weighting factors. *Journal of Research of the National Bureau of Standards*, 87(5):377–385.
